# Polymer/Titania Composites for the Remediation of Water from Pharmaceuticals: A Review

**DOI:** 10.1021/acsomega.5c09526

**Published:** 2026-01-29

**Authors:** Aliaksandr Kraskouski, Sarah Nealy, Eugenia Kharlampieva

**Affiliations:** † Department of Chemistry, 9968 The University of Alabama at Birmingham, Birmingham, Alabama 35294, United States; ‡ Center for Nanoscale Materials and Biointegration, The University of Alabama at Birmingham, Birmingham, Alabama 35294, United States

## Abstract

Clean water is a limited resource that is essential to human existence. Pharmaceuticals have been developed to improve the health and safety of living beings. Still, their widespread application and consumption, along with their increased industrial production and subsequent improper disposal, have resulted in unprecedented levels of pharmaceutical pollutants in water systems, adversely impacting the health of humans and aquatic and terrestrial organisms. Conventional wastewater treatment methods are ineffective in removing these persistent contaminants, leading to their distribution and accumulation in rivers, lakes, groundwater, and even drinking water sources. Therefore, the development of efficient, cost-effective, and sustainable materials for removing pharmaceuticals from wastewater is an immediate and growing necessity to maintain adequate water quality. Given the unique properties of polymers and the outstanding photocatalytic activity of titania (TiO_2_) nanomaterials, water treatment using polymer-based/TiO_2_ composites appears to be a promising approach. Although several reviews on the use of polymer/TiO_2_ composites for water treatment have been published, no comprehensive review has examined the performance of polymer/TiO_2_ composites, including natural, synthetic, and hybrid natural/synthetic polymer-based composites, in removing pharmaceuticals and addressed the key challenges associated with their practical applications. Herein, we provide an overview of polymer/TiO_2_ composites that are increasingly being explored for water purification and describe their performance in removing pharmaceuticals, including antibiotics, analgesics/anti-inflammatories, and other medical drugs commonly found in wastewater effluents. Key considerations, such as the toxicity of pharmaceutical degradation products, the reusability of polymer/TiO_2_ composites, and the impact of real-world water systems on overall treatment performance, are also discussed.

## Graphical abstract

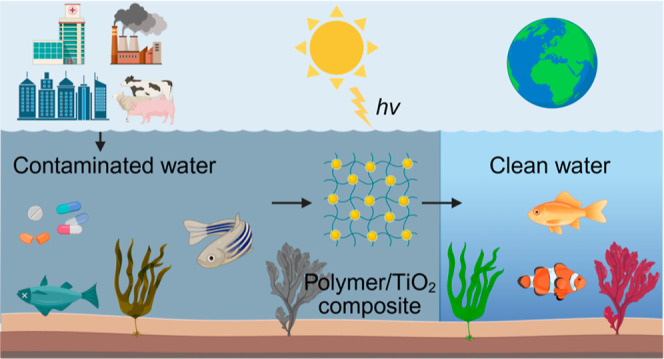

## 1 Introduction

Clean water is a limited resource that is essential to human existence. Freshwater reserves account for less than 3% of the world’s total water resources, and increasing pollution has made their preservation a global challenge. Humanity is facing drinking water scarcity exacerbated by the widespread discharge of untreated or insufficiently treated wastewater into water systems, as well as the ongoing depletion of natural freshwater resources. The continuous growth of the population is further reducing the availability of drinking water. Ensuring access to clean, safe water is central to the United Nations Sustainable Development Goals (SDGs), particularly SDG 6: Clean Water and Sanitation. By 2030, SDG 6 aims to improve water quality, ensure equitable and adequate sanitation and hygiene, and secure universal access to safe and affordable drinking water for all.^1^ Achieving these targets, however, is complicated by emerging threats to water quality posed by persistent contaminants such as pharmaceuticals. While pharmaceuticals have been developed to benefit public health, their widespread use and improper disposal have resulted in unprecedented levels of pharmaceutical contaminants in our water systems, negatively impacting the health of humans, aquatic organisms, and terrestrial organisms and contributing more broadly to climate change.^2–4^ Consequently, water pollution has emerged as a significant global concern that hinders sustainable development. The emergence of pharmaceuticals in water has prompted a range of serious environmental challenges, including bacterial resistance to antibiotics, the persistence of pharmaceuticals in aquatic ecosystems, and the bioaccumulation of drug residues in living organisms and marine environments, among others.^2,5–7^ Besides medical wastewater, municipal and livestock effluents contain unmetabolized pharmaceuticals, such as antibiotics, which pave the way for the progression of multidrug-resistant bacteria and waterborne pathogens.^3,8^ Among other interventions, the development of advanced treatment methods for removing pharmaceuticals from water is a matter of immediate concern. Pharmaceuticals are typically removed from wastewater in treatment plants through physical, chemical, and biological processes.^9–11^ However, these wastewater treatment processes do not eliminate pharmaceuticals from wastewater^11–14^ and are limited by shortcomings such as the formation of unwanted byproducts, high costs, and/or low removal efficiencies.^15,16^ Thus, the development of efficient, cost-effective, and sustainable materials for removing pharmaceuticals from wastewater is an urgent and growing necessity on a global scale that is essential for maintaining adequate water quality.

Hybrid polymeric nanomaterials are well-suited for the photocatalytic removal of pharmaceuticals from water and have shown considerable promise in addressing current challenges, thanks to their synergistic mechanical, electrical, and chemical properties.^17–19^ Given the versatility and unique structural and functional properties of polymers, along with the outstanding photocatalytic activity of titanium dioxide nanoparticles (TiO_2_ NPs), water treatment using polymer-based/TiO_2_ composites appears to be a promising strategy. In this regard, the photocatalytic degradation of pharmaceuticals by polymer/TiO_2_ composites has recently garnered increasing interest.

Several reviews have already been published regarding the use of polymer/TiO_2_ composites for water treatment. One review outlines the general principles and kinetics of heterogeneous photocatalysis, discussing the applications and photodegradation mechanisms of polymer/TiO_2_ composites for the photocatalytic removal of pharmaceuticals.^17^ A recent review focuses on the photocatalytic degradation mechanisms of TiO_2_-based materials, the different preparation routes of biopolymer/TiO_2_ photocatalysts, and the performances of various biopolymers, such as chitosan, cellulose, alginate, cyclodextrin, gum, starch, and collagen, as support materials for TiO_2_ during the removal of organic pollutants.^18^ Another review highlights advances in the development of biomaterial-based (chitosan, cellulose, and lignin)/TiO_2_ composite photocatalysts and their applications in removing trace pollutants, including heavy metals, dyes, and pathogenic microorganisms.^20^ Finally, another review summarizes the advancements in TiO_2_-based materials modified with conductive polymers, such as polyaniline, polypyrrole, and polythiophene, and outlines common strategies for modifying TiO_2_. It also describes the enhanced photocatalytic mechanism of polymer-modified TiO_2_ materials and their applications in the photocatalytic degradation of dyes and benzene derivatives.^21^

Still, open questions remain regarding the application of polymer/TiO_2_ composites for removing pharmaceuticals from water. First, the toxicity of pharmaceutical transformation products arising from photocatalytic degradation with polymer/TiO_2_ composites remains unclear and has not been thoroughly addressed. Second, the regeneration and removal efficiencies of polymer/TiO_2_ composites across multiple reuse cycles have not been fully discussed. Finally, the effect of real water systems with variable environmental conditions (e.g., salinity, pH, and organic matter) on pharmaceutical removal efficiencies remains underreported.

Here, we summarize the polymer/TiO_2_ composite materials widely used for removing pharmaceuticals from wastewater. We briefly present a classification of pharmaceuticals, their sources in water systems, and their primary methods of removal from water. Our review primarily focuses on composites based on natural and synthetic polymers, as well as their polymer hybrids, for the removal of water pollutants, encompassing a broad spectrum of pharmaceuticals, including antibiotics, analgesics, anti-inflammatories, and other medical drugs. Furthermore, the toxicity of pharmaceutical degradation products, the reusability of polymer/TiO_2_ composites, and the impact of real water systems on pharmaceutical removal performances are also considered.

## 2 Pharmaceuticals

Pharmaceuticals are chemicals commonly used to diagnose, treat, repair, or prevent diseases in both human health and veterinary medicine.^22,23^ They include a wide range of medications (Figure 1) that can be classified as antibiotics,^23^ analgesics and anti-inflammatory drugs,^22,24^ anticonvulsants,^25^ cardiovascular drugs,^26^ anticancer drugs,^27^ antidepressants,^28^ antiseptics,^29^ and antacids,^30^ among other medical drugs.^31^

**Figure 1:**
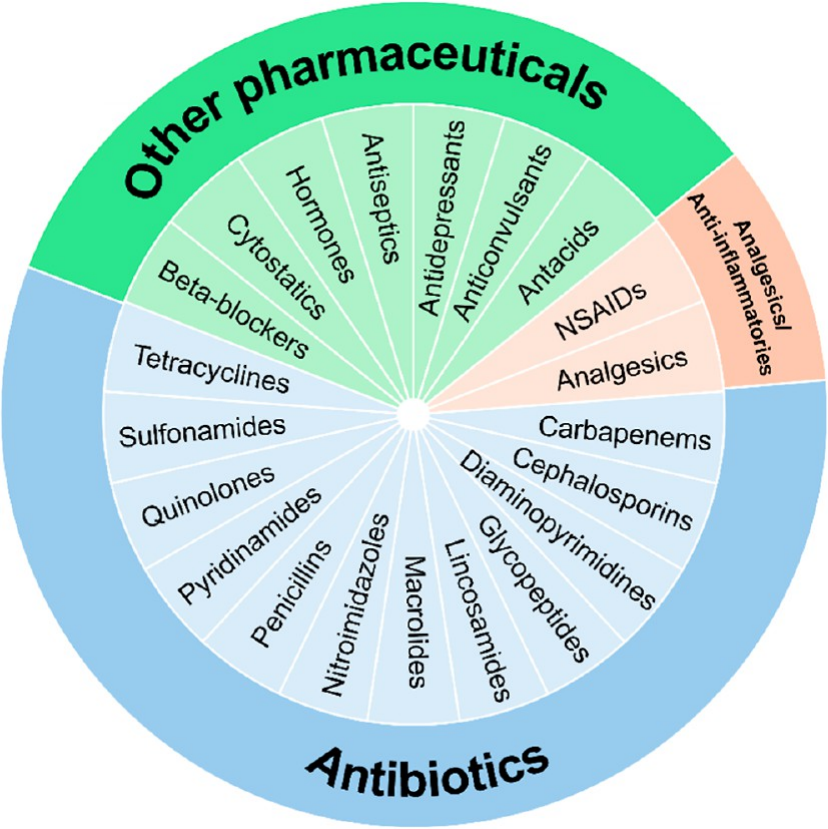
Different types of common pharmaceuticals.

Antibiotics are pharmaceuticals with a broad spectrum of antibacterial activity intended to treat and prevent bacterial infections. They are classified into aminoglycosides, macrolides, tetracyclines, glycopeptides, lincosamides, carbapenems, penicillins, cephalosporins, sulfonamides, and others (Figure 1, Table 1).^23^ Global antibiotic consumption was estimated at 49.3 billion defined daily doses in 2023 and is projected to increase by 52.3% by 2030.^32^ Several environmental challenges, including water pollution and the development of natural antimicrobial resistance, have accompanied their extensive usage.^33,34^

**Table 1:**
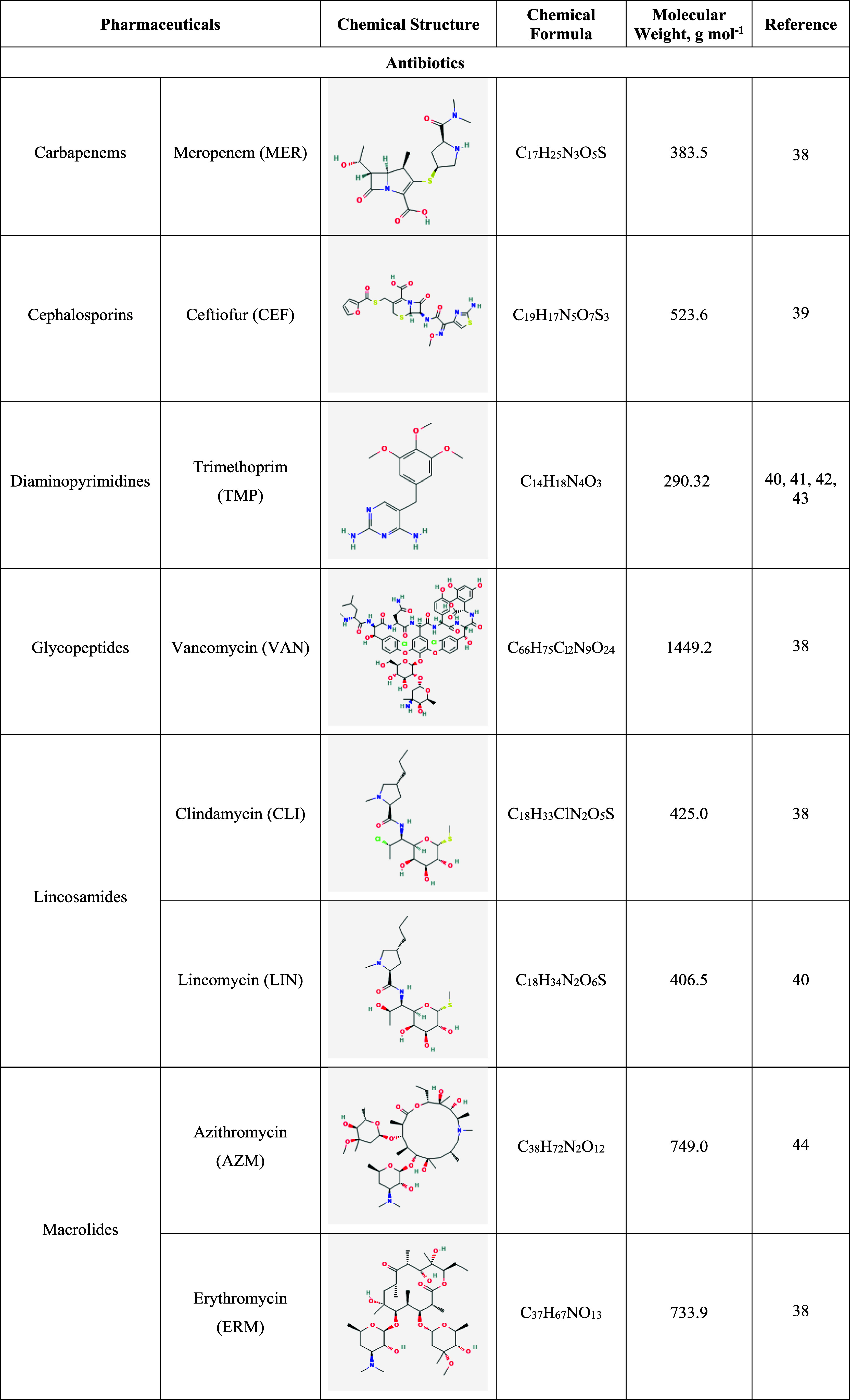

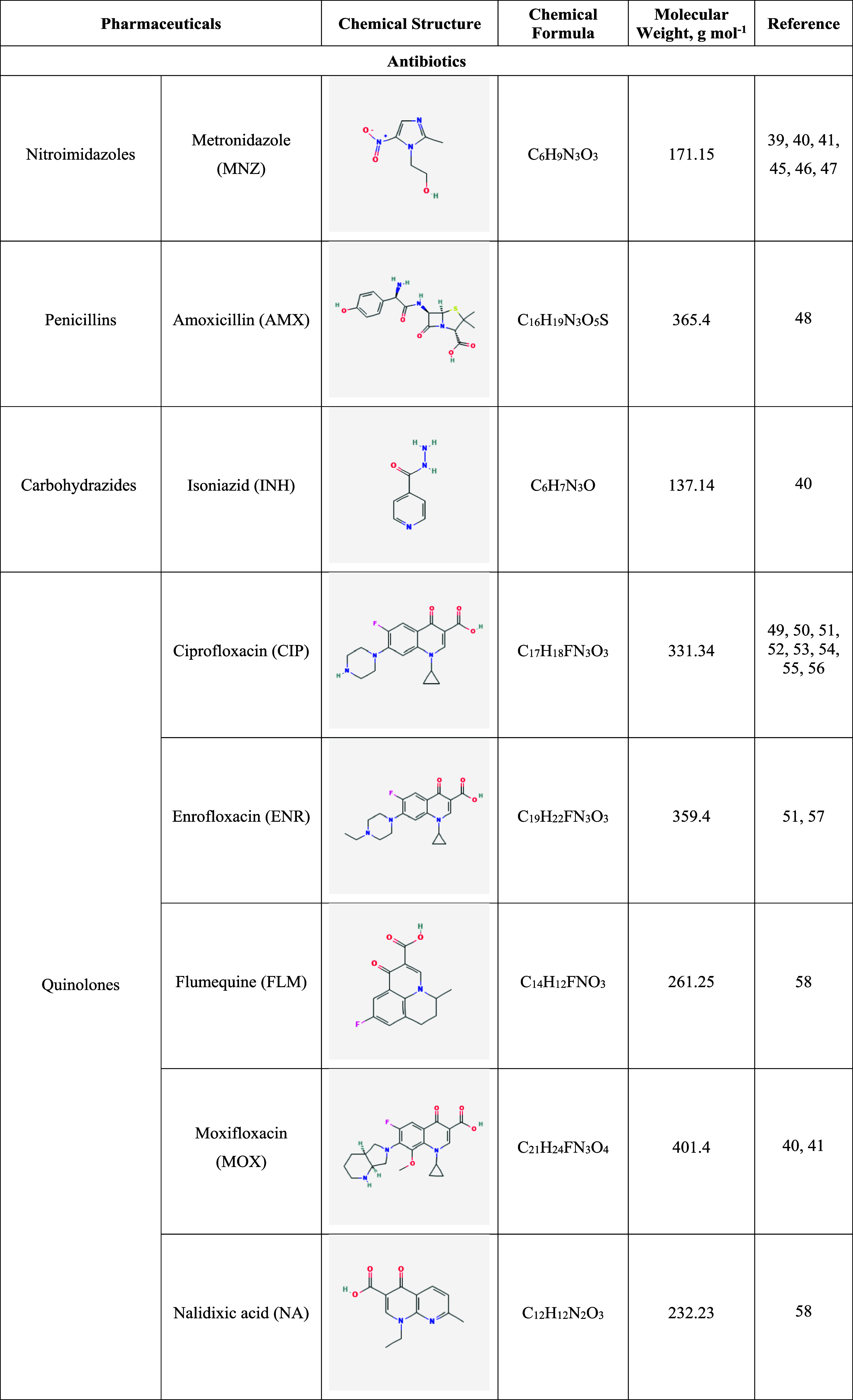

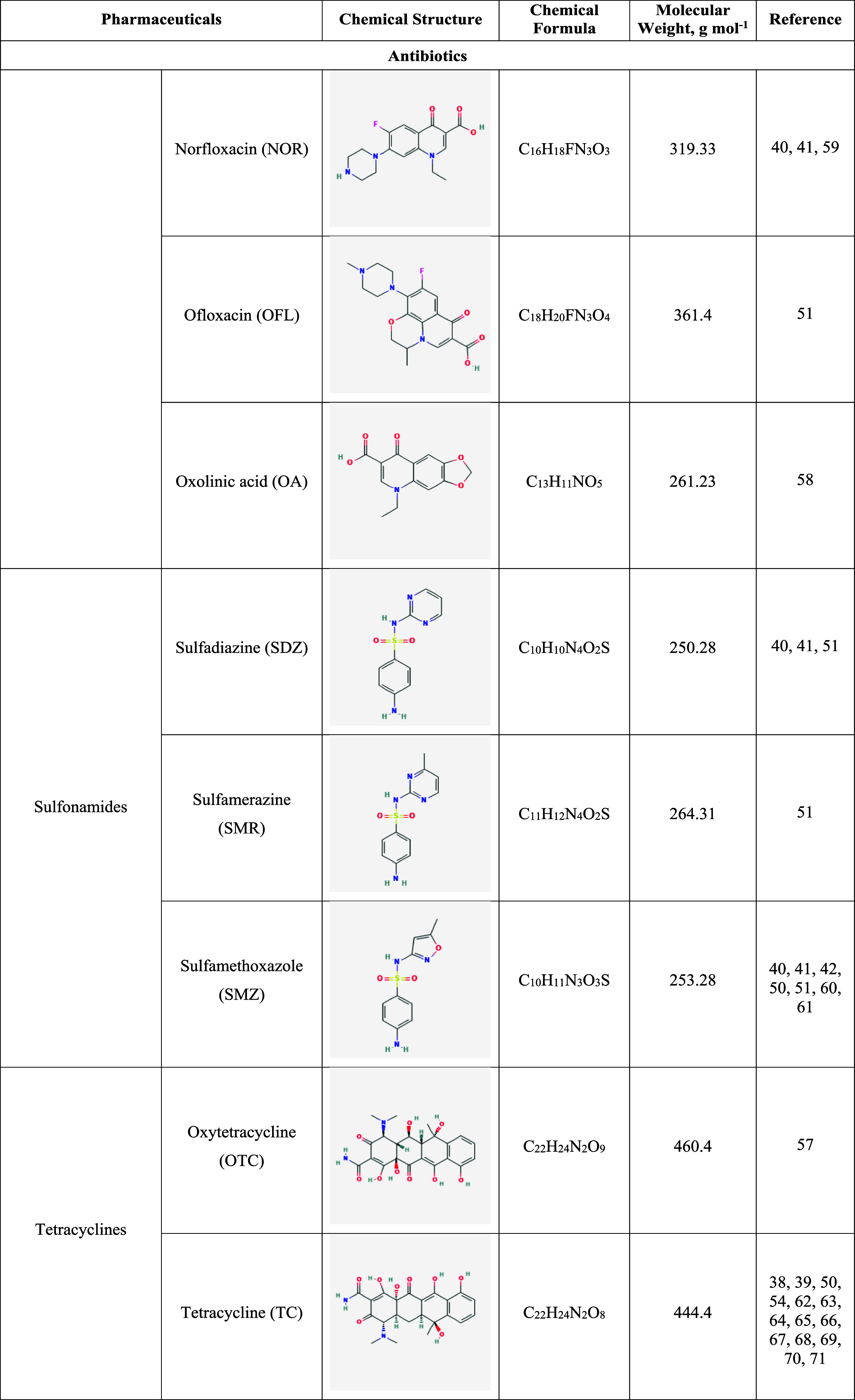

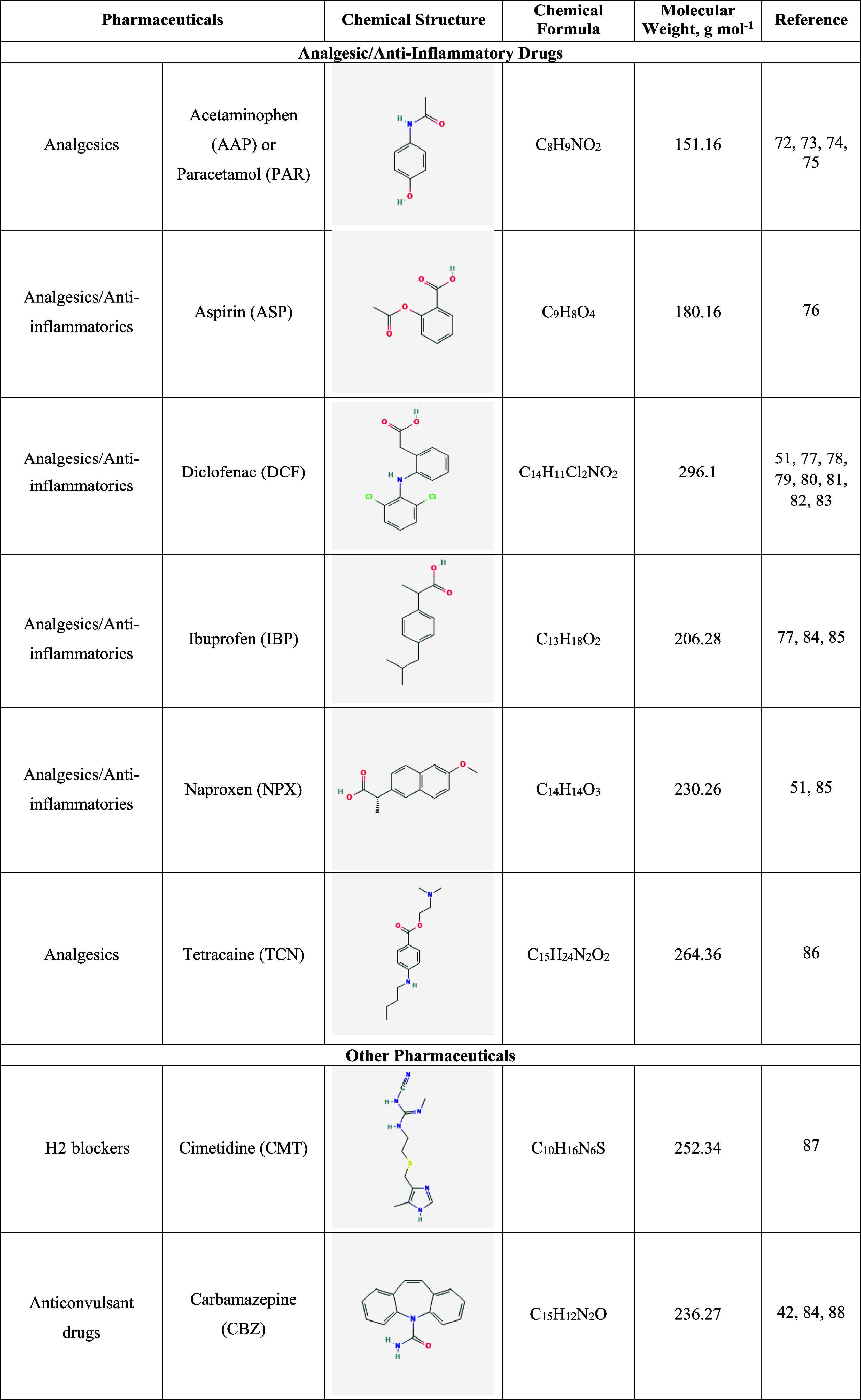

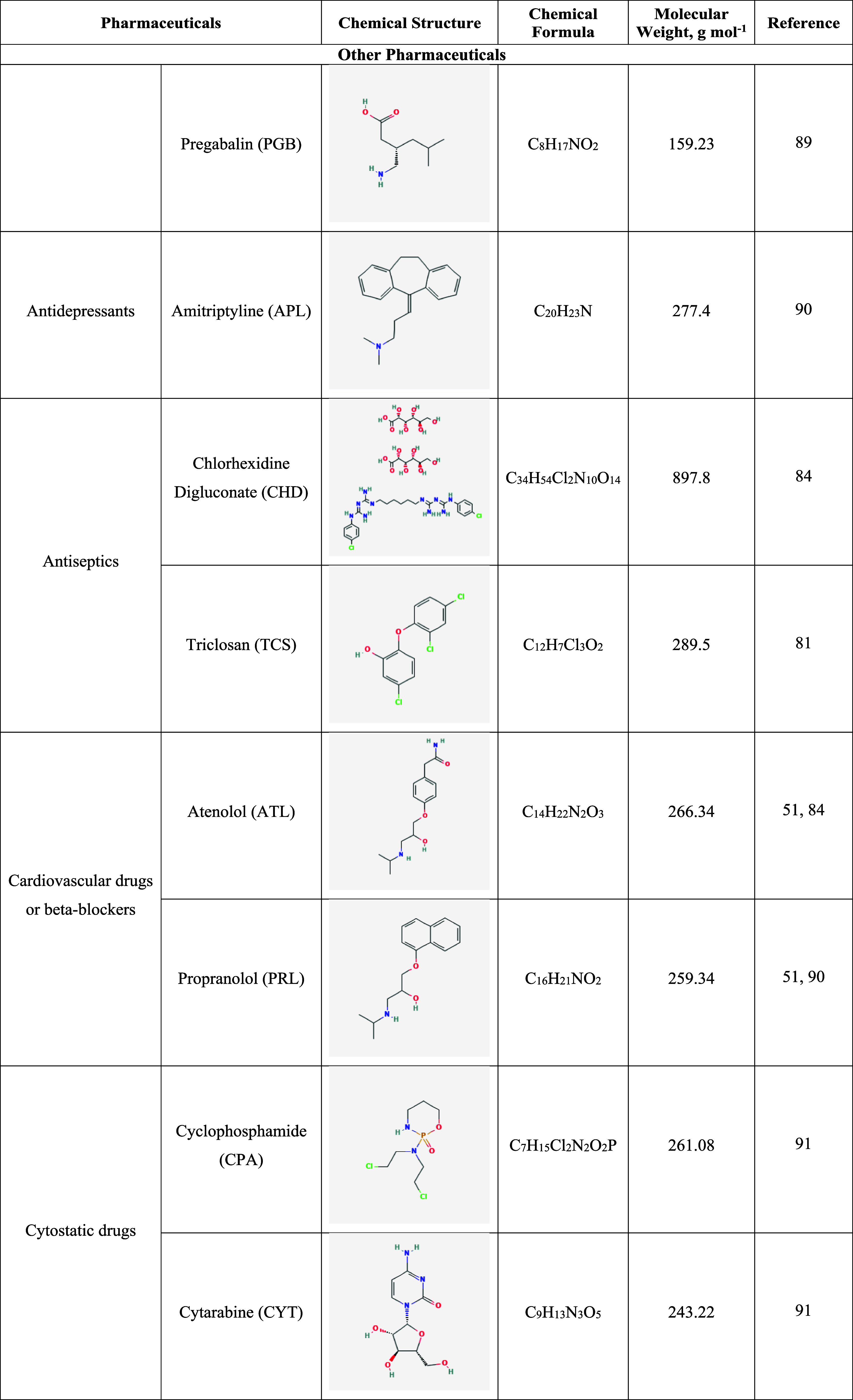

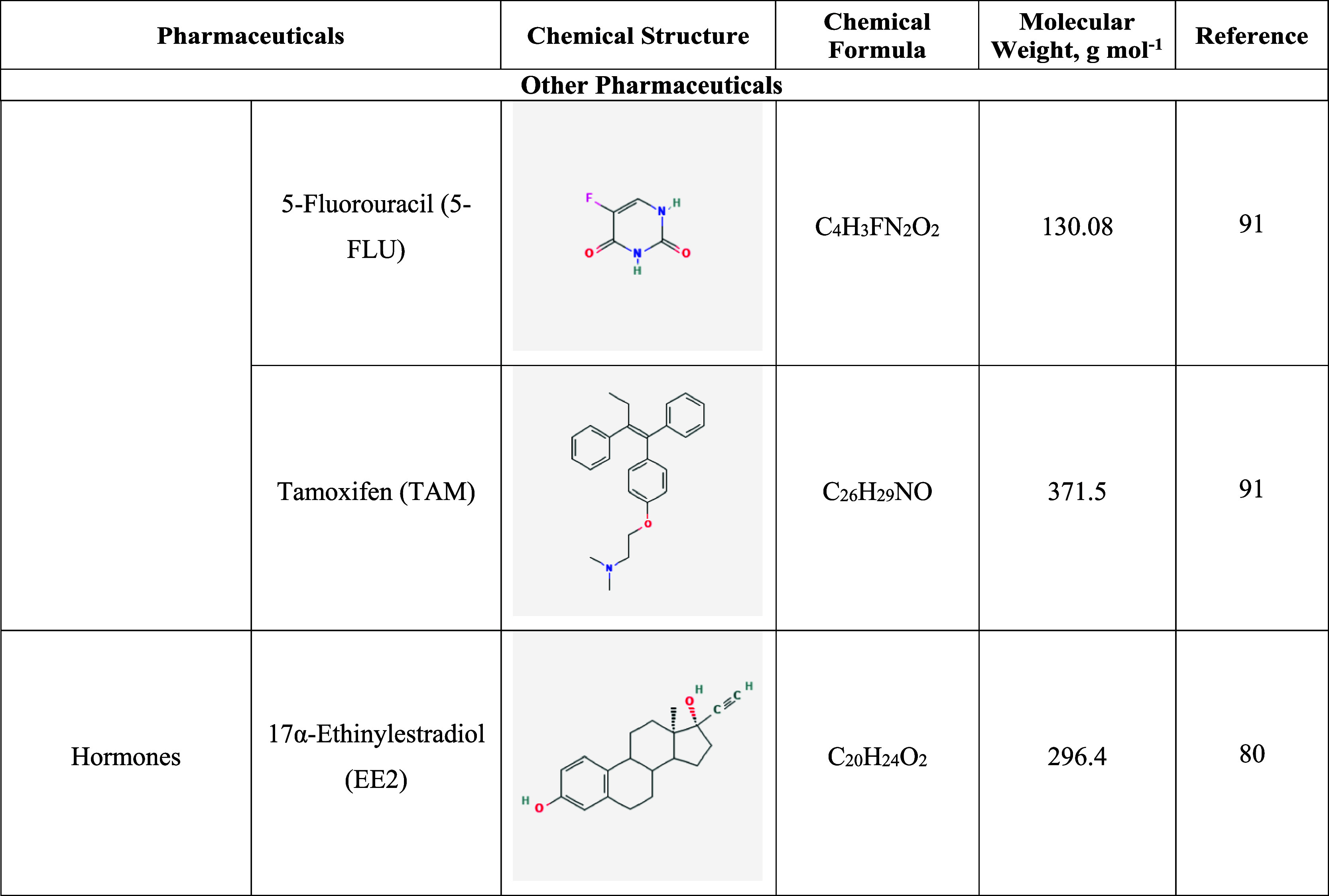
Descriptions of Pharmaceuticals That Are Considered in This Review^a^

a Chemical information on the pharmaceuticals was taken from PubChem.^92^ Pharmaceuticals are organized alphabetically.

Anti-inflammatory analgesics, also known as nonsteroidal anti-inflammatory drugs (NSAIDs), are the most consumed pharmaceuticals worldwide. They are frequently used in daily life to relieve pain, reduce inflammation, and lower fever.^24^ Many of these drugs, such as acetaminophen (Tylenol) and ibuprofen (Advil), can be accessed without a medical prescription, which has led to high levels of consumption. Their subsequent improper disposal and incomplete removal during wastewater treatment have resulted in the detection of many analgesic pharmaceuticals in surface water, plants, and edible crops.^35,36^

Table 1 summarizes the leading pharmaceuticals that are considered in this review. According to the IQVIA Institute report, the global use of pharmaceutical drugs was estimated at 3378 billion defined daily doses in 2023 and is expected to grow by 400 billion by 2028.^37^ The widespread consumption of pharmaceuticals, coupled with their increased production on an industrial scale, is a growing concern regarding the pollution of water systems, including rivers, lakes, oceans, and groundwater, as well as the management of these systems.^2,5^ Thus, designing green materials and new approaches for the efficient removal of pharmaceuticals from wastewater is a global challenge that is a primary necessity to address.

## 3 Sources of Pharmaceuticals in Water Systems

Pharmaceuticals have been found in wastewater influent and effluent, marine environments, drinking water, freshwater systems, and groundwater.^5,93,94^ The discharges from domestic sewage, pharmaceutical manufacturing facilities, aquaculture industries, medical and veterinary hospitals, and livestock farms are the primary sources where pharmaceuticals enter the water systems (Figure 2).^93,95^ The human body and that of livestock animals cannot completely metabolize pharmaceutical compounds, so drug residuals are excreted as urine, saliva, and feces and enter the sewage systems as influent wastewater.^96–98^ Additionally, flushing leftover medications down the drain contributes to their entry into municipal wastewater. The pharmaceutical manufacturing industry is a major contributor to pharmaceutical contamination in our aquatic environments.^99^ Moreover, the widespread use of pharmaceutical drugs to treat numerous diseases and infections in human and veterinary medicine has led to significant increases in pharmaceutical production and their consequential release into water systems. Pharmaceuticals are used in aquaculture to combat aquatic bacterial pathogens and may be added to feed and, therefore, are released directly into surface water.^100^ Hospitals, which use a wide range of medicines, including contrast media, analgesics, anti-inflammatory agents, and antibiotics, are another source of pharmaceuticals entering municipal wastewater systems.^101^ Pharmaceuticals from animal husbandry are recognized as a significant source of water pollution.^102,103^ Veterinary medicines are administered to treat and prevent various animal diseases, protect animals’ health, and promote weight gain. Their discharge into wastewater systems follows pathways similar to those of human pharmaceuticals. Hence, these multiple sources that release pharmaceuticals into the surrounding aquatic systems need to be monitored. Another strategy involves a more conservative, rational approach to the prescription and consumption of pharmaceuticals.

**Figure 2:**
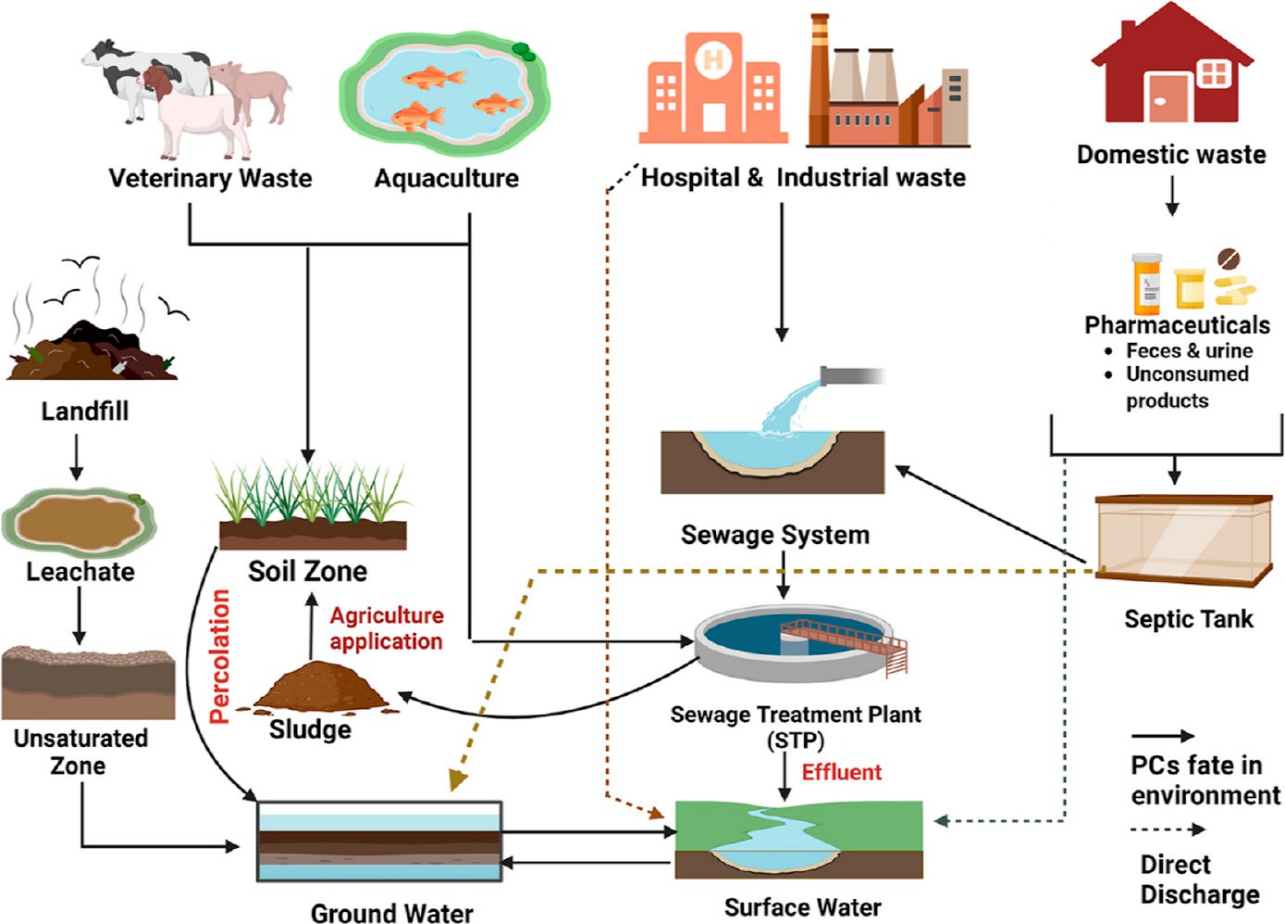
Potential sources and pathways for pharmaceutical contaminants entering water systems. Adapted with permission from the ref 2 Copyright 2023 American Chemical Society.

## 4 Main Strategies for Pharmaceutical Removal

Common approaches employed to remove pharmaceuticals from wastewater include physical collection (e.g., sedimentation, flocculation, membrane technology, and adsorption),^104–106^ chemical transformation (e.g., photocatalysis, chlorination, coagulation, and ozonation),^107–109^ biological processes (e.g., activated sludge, biosorption, and anaerobic digestion),^11,110,111^ and integrated/hybrid technologies (Figure 3).^112–114^ Among these, adsorption has proven to be an efficient method for removing pharmaceuticals due to its simplicity, absence of byproducts, and cost-effectiveness. At the same time, adsorptive materials have the added benefit of regeneration and reuse.^106,115,116^ However, adsorptive materials have certain drawbacks, such as diminished performance in heavily contaminated environments, limited adsorbent capacity, and high regeneration expenses, among other challenges.^106,117–119^ Photocatalysis is another promising approach for the remediation of water from pharmaceuticals, and the designs of photocatalytic materials can be complementary to adsorptive ones.^120,121^ The benefits of photocatalytic materials include high contaminant removal efficiency, easy maintenance, strong redox potential, cost-effectiveness, and enhanced durability compared to conventional sorbents.^19,122^ Still, certain limitations to these designs remain, such as insufficient penetration and absorption of visible light and high amounts of energy associated with their production.^117^ To address these challenges and increase pharmaceutical removal efficiencies, hybrid functional materials that combine the benefits of adsorption and photocatalysis are being explored.^123–126^ In this context, polymer-based composites with the “capture & destroy” effect show promise as hybrid materials for water treatment.^19,127^

**Figure 3:**
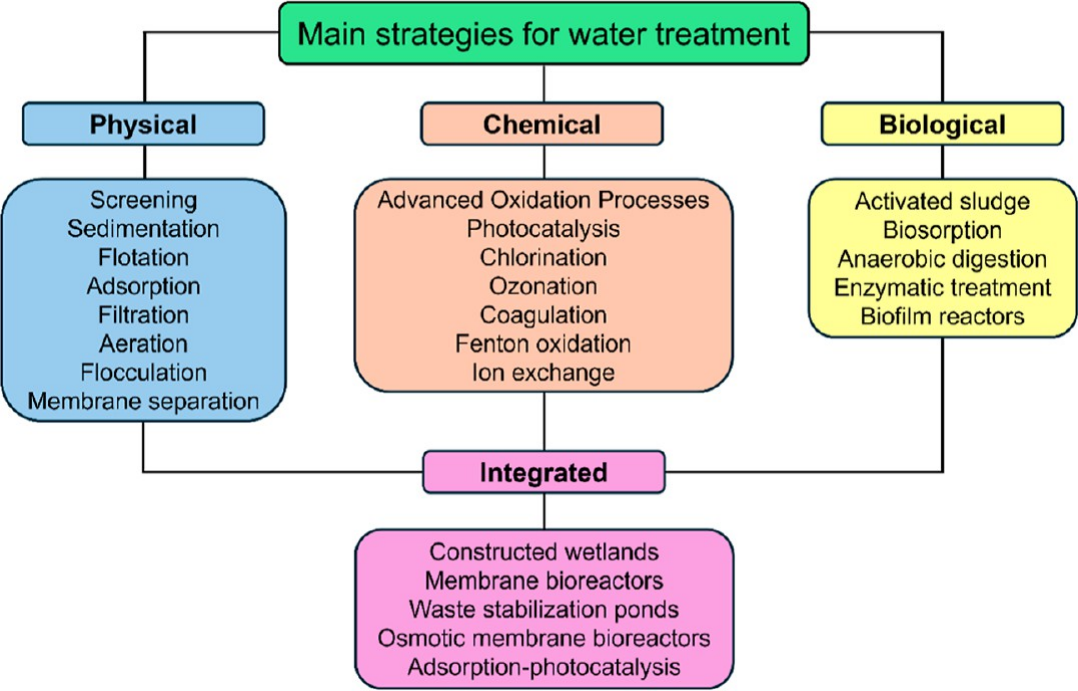
Main strategies for removing emerging contaminants from wastewater.

## 5 Titanium Dioxide as a Photocatalyst

Titanium dioxide is a versatile nanomaterial and one of the most widely used photocatalytic semiconductors due to its outstanding physical and chemical properties. It has been widely utilized for photovoltaic applications,^128,129^ hydrogen production,^130,131^ conversion of CO_2_,^132,133^ energy storage,^134,135^ gas sensing,^136,137^ textile industry,^138,139^ biomedical applications,^140,141^ self-cleaning applications,^142,143^ food industry,^144^ cosmetics,^145^ and water treatment.^146–149^

Among the photocatalysts studied in water treatment, TiO_2_ is widely appealing due to its high photocatalytic activity, high specific surface area, excellent chemical stability, and relatively low production cost compared to other metal oxides (Table 2).^108^ TiO_2_ exists in one of three crystalline phases: anatase, rutile, and brookite. Generally, anatase TiO_2_ is considered a more effective photocatalyst compared to rutile TiO_2_.^150,151^ However, the mixture of anatase and rutile TiO_2_ phases has shown increased photoactivity compared to monocrystalline anatase TiO_2_ due to structural defects embedded by the rutile crystalline phase that promote charge transfer and extend the longevity of electron–hole pairs generated by the anatase phase.^152–155^

**Table 2:** Main Advantages and Disadvantages of TiO_2_ for Practical Applications

main advantages	main disadvantages/limitations
high photocatalytic activity	low absorption of visible light
chemical stability	poor affinity to hydrophobic contaminants
high surface area	high aggregation tendency
relatively low cost for production	difficulty of separation and recovery
availability	toxic effects^a^
excellent durability	
antimicrobial activity	
nontoxicity	

a TiO_2_ NPs may pose toxicity risks due to their environmental persistence if not properly managed.

However, TiO_2_ has disadvantages that limit its practical utility, including low visible-light absorption, poor affinity for hydrophobic contaminants, high aggregation tendency, and difficulty in separation and recovery (Table 2). Several strategies have been proposed to overcome these shortcomings and expand the prospects of TiO_2_, including doping, coupling, surface organic modification, heterojunction design, dye-sensitization, and structural modification, among others.^156–160^ Nevertheless, using TiO_2_ as a powder requires energy-consuming separation steps to remove it at the end of the water treatment process. Furthermore, the persistence of TiO_2_ NPs in the environment raises concerns about potential toxicity if not appropriately managed.^161–165^ TiO_2_ NPs must be removed from the treated water to prevent secondary water contamination and mitigate harmful effects on ecosystems and the organisms living in them. Among the suggested approaches, encapsulation, immobilization, or capping polymer substrates are considered effective strategies.^18,20,127,166^ The development of hybrid organic-TiO_2_ nanomaterials introduces an approach that affords tunable chemistry, and this design feature has been leveraged for the controlled growth and phase development of crystalline TiO_2_ on reactive organic surfaces.^152,154,167–170^ The integration of polymers, possessing distinct physical and chemical properties, with photocatalytic TiO_2_ nanomaterials represents a promising strategy to prevent secondary water contamination by TiO_2_, thus mitigating the harmful effects of metal oxide NPs on ecosystems, improving contaminant removal efficiency, preventing aggregation in aqueous media, and simplifying the post-treatment TiO_2_ separation process.

## 6 Polymer/TiO_2_ Composite Materials for Pharmaceutical Removal

Polymer-based/TiO_2_ composites are prospective materials for water remediation due to their superior properties, including mechanical strength, durability, tunability, ease of handling, and adaptability.^17–21,171,172^ These materials possess improved stability and reusability compared to traditional ones, making water treatment more energy- and cost-effective. The tunable surface functionalities of polymers enable tailored interactions with various hydrophobic and hydrophilic, as well as anionic and cationic, water contaminants, thereby enhancing selectivity and removal performances. Polymer/TiO_2_ composite materials based on conductive polymers exhibit enhanced photocatalytic activity due to photosensitization and the synergistic effect between the polymer and TiO_2_.^21^ Additionally, the polymer support prevents the agglomeration of TiO_2_ NPs and simplifies their post-treatment separation. Moreover, the versatility and tunability of polymers provide the fabrication of composites in diverse forms (e.g., films, membranes, beads, fibers, particles, etc.) to meet specific application requirements. Overall, water remediation using polymer/TiO_2_ composites offers a sustainable and reliable approach.

### 6.1 Natural Polymer/TiO_2_ Composites

#### 6.1.1 Natural Polymers

Nowadays, many natural polymers are widely used in water treatment.^19,173^ Natural polymers are derived from renewable sources in the environment (Figure 4). Among them, polysaccharides, such as cellulose, chitosan, and alginate, have gained increasing attention as materials for water treatment due to their biocompatibility, biodegradability, accessibility, renewability, and nontoxicity (Figure 4).^174,175^

**Figure 4:**
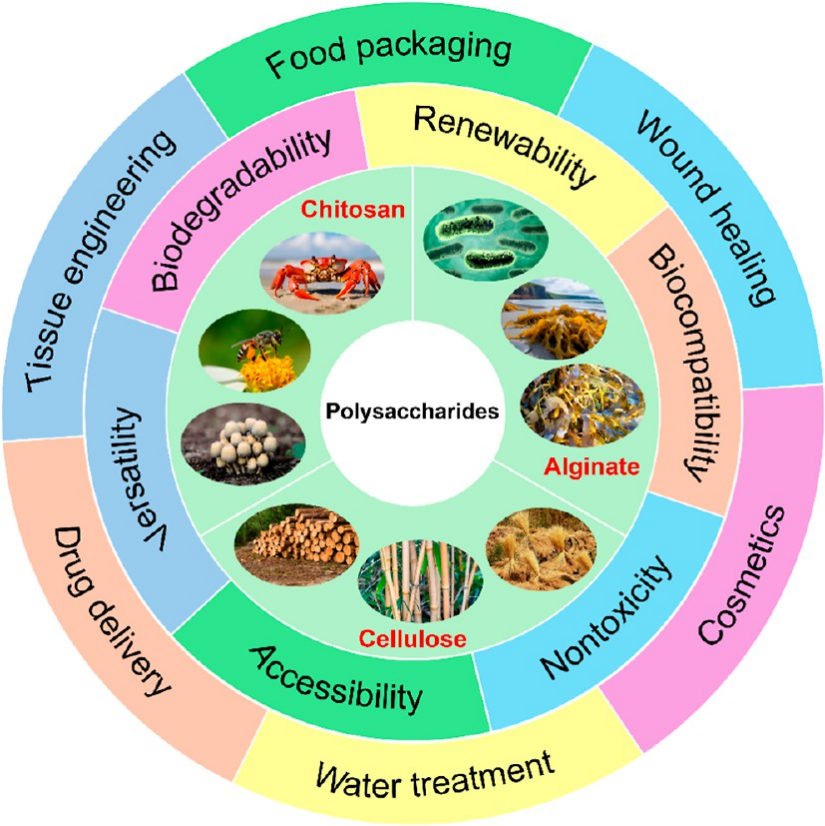
Schematic illustration of sources, unique properties, and applications of polysaccharides.

Cellulose is the most abundant naturally occurring polysaccharide, consisting of a linear chain of d-glucopyranose units joined by β-(1 → 4)-glycosidic bonds. The primary sources of cellulose are plants, wood, grass, and agricultural wastes (Figure 4).^176^ This biopolymer demonstrates potential for use in food packaging,^177^ wound healing,^178^ nutraceutical^179^ and biomedical^180^ applications, as well as wastewater treatment.^181^ The widespread use of cellulose in various applications is mainly due to its biocompatibility, biodegradability, abundant reserves, water-retention ability, adjustable rheological properties, mechanical properties, and flexibility.^180,182^

Chitosan, being the second most abundant natural biopolymer after cellulose, is a versatile cationic aminopolysaccharide of animal or fungal origins (Figure 4) composed of randomly distributed β-(1 → 4)-linked d-glucosamine and *N*-acetyl-d-glucosamine units.^183^ Chitosan is a unique biopolymer that is generally recognized as safe, and due to its remarkable properties, such as biodegradability, biocompatibility, nontoxicity, sorption ability, antifungal, antioxidant, and antimicrobial activities, it has emerged as a promising and suitable biopolymer for potential biomedical,^184,185^ food packaging,^186^ cosmetics,^187^ and environmental^188^ applications.

Alginate is a natural anionic polysaccharide isolated from brown algae, such as *Laminaria digitata* and *Ascophyllum nodosum* (Figure 4). It is comprised of β-d-mannuronic acid and α-l-guluronic acid linked by (1 → 4)-glycosidic bonds.^189^ Alginate is characterized by excellent hydrophilicity, gelation ability, fast gelation, low cost, and ease of processing. It is a nontoxic, biocompatible, biodegradable, and renewable polysaccharide with a wide range of applications, including food packaging,^190^ tissue engineering,^191^ wound therapy,^192^ and water treatment.^189,193^

#### 6.1.2 Antibiotic Removal

Numerous scientific papers have recently reported the combination of TiO_2_ with natural polymers as supporting substrates for antibiotic removal from water (Table 3). Among the natural polymer/TiO_2_ composites, those based on cellulose have gained increasing attention. For example, Wang et al. developed a novel and facile electrospinning-electrospray (EE) method based on the electrospinning technique and simultaneous electrospray (Figure 5A) and prepared rice straw-derived cellulose acetate/TiO_2_ nanofibrous membranes (EE-CA/P25) with different TiO_2_ dosages (0.03, 0.05, and 0.10 g).^50^ After 30 min of visible light exposure, the EE-CA/P25_(0.05)_ membranes exhibited photocatalytic degradation efficiencies of 83.59%, 51.48%, and 14.25% for TC, CIP, and SMZ antibiotics, respectively. Moreover, EE-CA/P25_(0.05)_ demonstrated a high antimicrobial efficiency of 98.42% against *E. coli*. In another study, Huang et al. fabricated an alkali-cooking modified rice straw fiber (AMSF) coated with a TiO_2_ hydrogel layer (TiO_2_@AMSF) to remove CIP from water.^49^ The CIP removal capacities by TiO_2_@AMSF were 153, 112, and 60 mg g^–1^ under UV, natural light, and darkness, respectively. However, the CIP removal in a mixture of CIP, MOX, and levofloxacin hydrochloride under natural light was cut in half. Zeghioud et al. investigated the photocatalytic degradation of other quinolone antibiotics (FLM, OA, and NA) commonly used in aquaculture under UV-A light irradiation using TiO_2_ impregnated on cellulosic paper (CF/TiO_2_) as a supported photocatalyst.^58^ Complete degradation was observed for all antibiotics within 4 h. FLM, OA, and NA degradation efficiencies in the ternary mixture were about 95%, 75%, and 90%, respectively, within 5 h. Other authors fabricated a TiO_2_-containing nanocomposite aerogel (C-SLCNF-TNPs) based on sulfated lignocellulose nanofibrils (SLCNF), extracted from bagasse via a deep eutectic solvent-based approach and used it for adsorptive–photocatalytic synergistic removal of TC (Figure 5B).^62^ The maximum adsorption capacity of TC was 70 mg g^–1^ (35% removal) after 80 min, while the photocatalytic degradation rate of TC reached 59.9% within 120 min, revealing a high total adsorption-degradation removal efficiency of 94.9%.

**Table 3:** Natural Polymer/TiO_2_ Composites for Antibiotic Removal

composite	target pharmaceuticals	composite form	irradiation	reusability	reference
antibiotics					
cellulose acetate/TiO_2_ (EE-CA/P25)	CIP, SMZ, TC	membrane	VISIBLe light	N/A	^50^
alkali-cooking modified rice straw fiber/TiO_2_ (TiO_2_@AMSF)	CIP	fibers	UV, natural light, dark	3 cycles	^49^
cellulose fibers/TiO_2_ (CF/TiO_2_)	FLM, NA, OA	fibers	UV light (365 nm)	N/A	^58^
lignocellulose/TiO_2_ (C-SLCNF-TNPs)	TC	aerogel	UV light (365 nm)	5 cycles	^62^
ethyl cellulose/TiO_2_ (TiO_2_@EC)	TC	powder	N/A	5 cycles	^64^
cellulose nanocrystals/TiO_2_ (CNC–TiO_2_)	AZM	powder	direct sunlight	N/A	^44^
chitosan/TiO_2_ (CS/TiO_2_)	CLI, ERM, MER, TC, VAN	membrane	visible light	N/A	^38^
chitosan/TiO_2_ (CS–TiO_2_)	MNZ	beads	UV light (254 nm)	15 cycles	^45^
chitosan/TiO_2_ (TiO_2_/CS)	AMX	scaffold	UV/vis (300–800 nm)	3 cycles	^48^
chitosan/TiO_2_ (CS@TiO_2_)	TC	film	UV light (360 nm)	4 cycles	^69^
chitosan/N, S-doped TiO_2_ (NST/CS)	TC	powder	visible light	4 cycles	^70^
alginate/chitosan/TiO_2_ (mAL/CH/TiO_2_)	TC	beads	N/A	3 cycles	^66^
EPI-cross-linked β-cyclodextrin/TiO_2_ (β-CDP/TiO_2_)	TC	powder	visible light	5 cycles	^65^
cyclodextrin-epichlorohydrin copolymer/TiO_2_ (β-EPI-TiO_2_)	SMZ	particles	UV light (254 nm)	N/A	^60^
keratin/TiO_2_ (K–TiO_2_)	TMP	hydrogel	simulated sunlight	4 cycles	^43^

**Figure 5:**
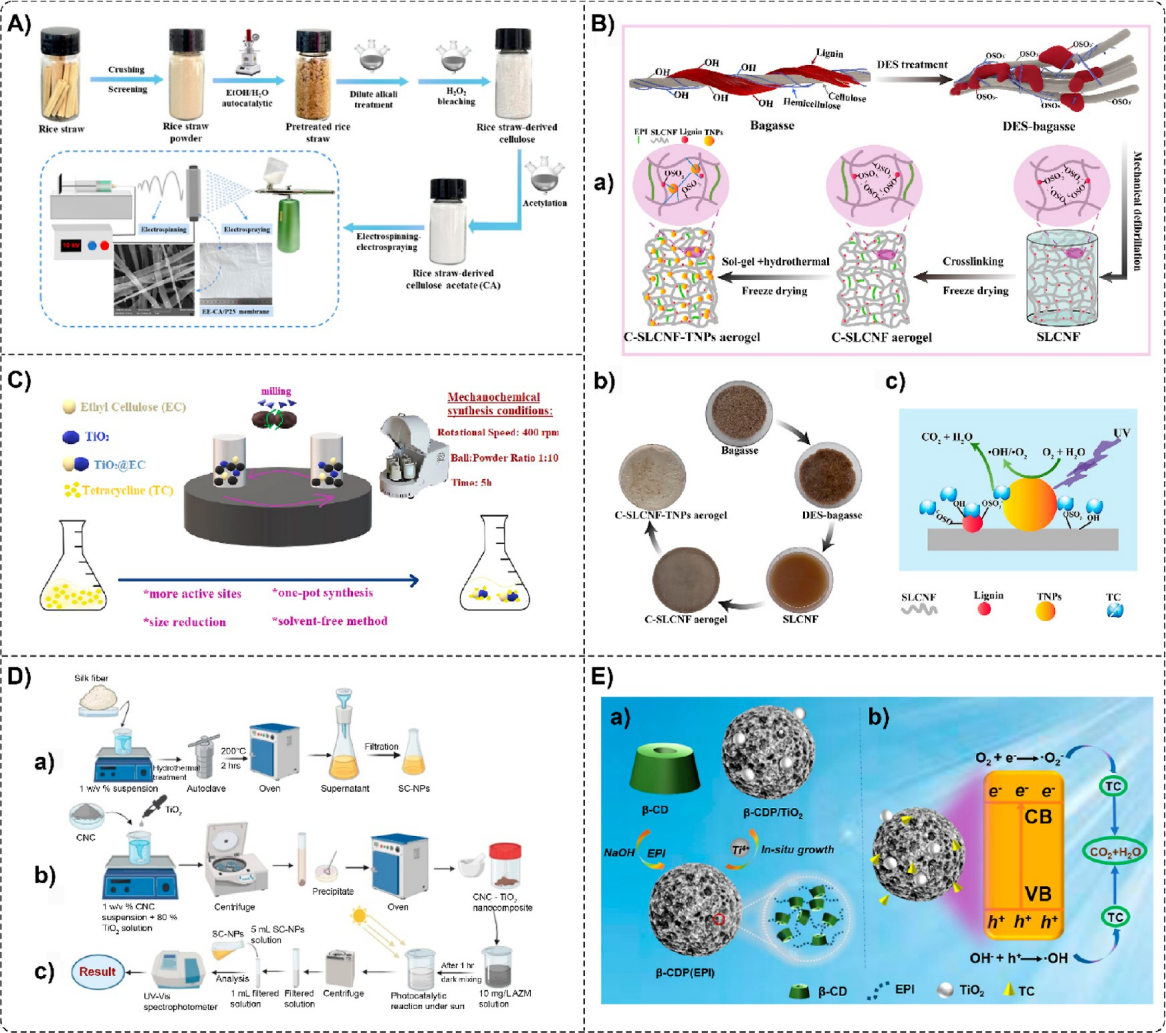
(A) Schematic illustration of the EE-CA/P25 nanofiber membrane fabrication process. Reprinted from the ref 50 Copyright (2024), with permission from Elsevier. (B) Schematic illustration of C-SLCNF-TNPs aerogel preparation (a), visual appearance of bagasse, deep eutectic solvent (DES)-bagasse, SLCNF, C-SLCNF aerogel, and C-SLCNF-TNPs aerogel (b), and mechanism of TC adsorptive–photocatalytic removal using C-SLCNF-TNPs aerogels (c). Reprinted from the ref 62 Copyright (2022), with permission from Elsevier. (C) Synthesis of TiO_2_@EC composites by the mechanochemical method. Reprinted from the ref 64 Copyright (2023), with permission from Elsevier. (D) Schematic overview of the experimental process for synthesis of silk-derived carbon NPs (SC-NPs) (a), fabrication of CNC–TiO_2_ nanocomposites (b), and photocatalytic treatment of AZM and quantification of AZM removal (c). Adapted with permission from the ref 44 Copyright 2024 Springer Nature. (E) Schematic illustration of the β-CDP/TiO_2_ composite fabrication (a) and TC photocatalytic degradation (b) processes. Reprinted from the ref 65 Copyright (2024), with permission from Elsevier.

Kanmaz et al. also investigated the removal of TC from aqueous solutions using a cellulose-based/TiO_2_ composite.^64^ In this study, a hybrid composite (TiO_2_@EC) was synthesized via mechanochemical ball-milling by adding different amounts of TiO_2_ to ethyl cellulose (Figure 5C). The maximum TC adsorption capacity was found to be 23.26 mg g^–1^ for 80%TiO_2_@EC. Meanwhile, Saha and Varanasi explored a cellulose-based/TiO_2_ composite [cellulose nanocrystal (CNC)–TiO_2_] for the removal of another class of antibiotics, macrolides.^44^ In this work, the authors synthesized photocatalytic nanocomposites with high loading (80 wt %) of TiO_2_ NPs on the surface of the CNC scaffold (20 wt %) using a solution precipitation method (Figure 5D). A maximum AZM removal efficiency of 98.8% was reached in 5 h under direct sunlight.

In addition to cellulose-based composites, antibiotics may be effectively removed from water using chitosan-based ones. For example, Spoială et al. fabricated chitosan/TiO_2_ (CS/TiO_2_) composite membranes using a simple casting method, followed by glutaraldehyde cross-linking and lyophilization.^38^ The removal efficiencies of a five-antibiotic (50 ppb CLI, TC, ERM, VAN, and MER) mixture during 48 h under visible light irradiation with CS/TiO_2_ 5% membranes were over 98% for TC and MER, while those values for CLI, VAN, and ERM were 68%, 86%, and 88%, respectively. Moreover, CS/TiO_2_ composite membranes exhibited pronounced antibacterial activity against *E. coli* and *Citrobacter* species.

In another study, Neghi et al. synthesized chitosan/TiO_2_ (CS–TiO_2_) composite beads (1.5–3 mm) via precipitation in an alkali/solvent medium and investigated their photocatalytic activity toward MNZ.^45^ Under UV light, 98.2% antibiotic removal was observed. At the same time, Bergamonti et al. proposed TiO_2_-supported chitosan scaffolds (TiO_2_/CS), designed by 3D printing, followed by ionotropic gelation, for the AMX photodegradation under UV–vis irradiation.^48^ Complete AMX degradation was observed after about 3 h of irradiation. In another study, Ikhlef-Taguelmimt et al. investigated TiO_2_-immobilized chitosan (CS@TiO_2_) films obtained using the casting/cross-linking method.^69^ The removal efficiency of TC under UV irradiation was 87% using 0.12 g of TiO_2_ in films with a chitosan:TiO_2_ ratio of 2:1. TC removal was significantly increased by agitation of the solution, increasing from 25% without agitation to 72% with agitation within a 60 min reaction time. For the degradation of TC under visible light irradiation, Farhadian et al. proposed an N, S-doped TiO_2_/chitosan (NST/CS) nanocomposite. The NST/CS composites were synthesized by a sol–gel-hydrothermal method.^70^ The NST/CS catalyst achieved an adsorptive TC removal of 25% within 60 min and a photocatalytic degradation efficiency of 91% within 20 min under visible light. Rizzi et al. used two natural polymers, alginate and chitosan, to synthesize alginate/chitosan/TiO_2_ (mAL/CH/TiO_2_) microbeads via external gelation, followed by immersion in chitosan solution for adsorptive removal of TC from water.^66^ After 180 min, about 50% of the TC was removed. The maximum adsorption capacity was found to be 0.96 mg g^–1^. Zhang et al. used another natural polymer-based/TiO_2_ nanocomposite for TC removal.^65^ The authors prepared a novel epichlorohydrin (EPI)-cross-linked β-cyclodextrin polymer/TiO_2_ (β-CDP/TiO_2_) composite (Figure 5E). The β-CDP/TiO_2_ composite exhibited a 96% degradation efficiency of TC under solar light after 90 min, which was 2.5 times higher than that of TiO_2_. A similar β-cyclodextrin-epichlorohydrin copolymer/TiO_2_ nanocomposite (β-EPI-TiO_2_) was investigated by Rizzi et al. for the removal of SMZ from water by an adsorption process, followed by an advanced oxidation process for the adsorbent’s regeneration.^60^ After 60 min of contact time, 39% of SMZ was removed via adsorption using β-EPI-TiO_2_ containing 12.5 mg of TiO_2_ per gram of cyclodextrin, which was lower than that of β-EPI alone (52%). Furthermore, under UV irradiation, β-EPI-TiO_2__12.5 achieved 81% removal of adsorbed SMZ, compared to 77% for β-EPI. Therefore, the use of β-EPI-TiO_2_ nanocomposites did not significantly enhance the efficiency of the advanced oxidation process, even under continuous UV irradiation, and instead reduced the β-EPI adsorption capacity.

Among natural biopolymers, proteins have also been studied for water treatment applications. For example, Villanueva et al. developed keratin hydrogels with immersed TiO_2_ NPs (K–TiO_2_).^43^ These hydrogels exhibited the ability to adsorb and degrade TMP under simulated solar light. The adsorption capacity after 18 h of contact ranged between 0.10 and 1.50 mg g^–1^ at an adsorbent dosage of 0.01 g L^–1^ and an initial antibiotic concentration of 0.172 mM. The TMP removal was approximately 50% within 2 h of light exposure, and a *C*/*C*_0_ value of around 0.20 was achieved for K–TiO_2_ 10% after 7 h of interaction time.

#### 6.1.3 Analgesic/Anti-Inflammatory Drug Removal

In addition to antibiotics, the removal of analgesics and anti-inflammatories using natural polymer-based TiO_2_ composites is being actively studied (Table 4). For example, Dionmbete et al. fabricated multifunctional lignocellulosic biocomposites from agricultural palm fiber waste.^83^ The raw palm fibers were activated by gliding an arc nonthermal plasma that modified their surfaces by grafting –OH and –COOH groups that served as anchoring sites for TiO_2_ immobilization (Figure 6A). The synthesized biomass/TiO_2_ (LCB/TiO_2_) biocomposites were explored for the removal of DCF from water. The maximum uptake capability of DCF (50 mg L^–1^) at pH 5, 100 min of contact time, and 25 °C was found to be 37.91 mg g^–1^. The LCB/TiO_2_ biocomposite (0.5 g L^–1^) exhibited a DCF removal efficiency of 99.75% within 60 min of treatment under gliding arc irradiation. In another study, Jallouli et al. used cellulosic fibers/TiO_2_ (TiO_2_–CF) composites to investigate the photocatalytic degradation of PAR, an analgesic drug, under direct sunlight.^74^ The degradation efficiency of PAR was 83% within 150 min. This study showed that the TiO_2_–CF/sunlight system was less effective than the TiO_2_ suspension/UV system for the photocatalytic degradation of PAR. Still, the former allows for easy separation of the photocatalyst from purified water. Another cellulose-based/TiO_2_ composite was also reported to degrade AAP. Lucchini et al. prepared hybrid cellulose nanofibril (CNF)/TiO_2_ monoliths (98% porosity) using CNFs impregnated with TiO_2_ NPs and water as a solvent (Figure 6B).^75^ The compression tests revealed a good shape recovery of the CNF/TiO_2_ monolith after unloading. This composite material was explored for the decomposition of PAR. The results showed that 30% of PAR (20 ppm solution) degraded after 150 min under in-flow solar illumination, reaching 51% after 500 min.

**Table 4:** Natural Polymer/TiO_2_ Composites for Removal of Analgesic/Anti-Inflammatory and Other Pharmaceuticals

composite	target pharmaceuticals	composite form	irradiation	reusability	reference
analgesics					
lignocellulosic biomass/TiO_2_ (LCB/TiO_2_)	DCF	powder/particles	gliding arc plasma	5 cycles	^83^
cellulose/TiO_2_ (TiO_2_–CF)	PAR	fibers	direct sunlight	5 cycles	^74^
cellulose/TiO_2_ (CNF/TiO_2_)	PAR	nanofibril monolith	simulated sunlight	N/A	^75^
alginate/TiO_2_ (TIAB)	IBP	beads	UV light (365 nm)	N/A	^84^
alginate/TiO_2_ (Alg/TiO_2_)	DCF, TCN	beads	UV light (365 nm)	N/A	^86^
other pharmaceuticals					
alginate/TiO_2_ (TIAB)	CBZ, ATL, CHD	beads	UV light (365 nm)	5 cycles for CHD	^84^

**Figure 6:**
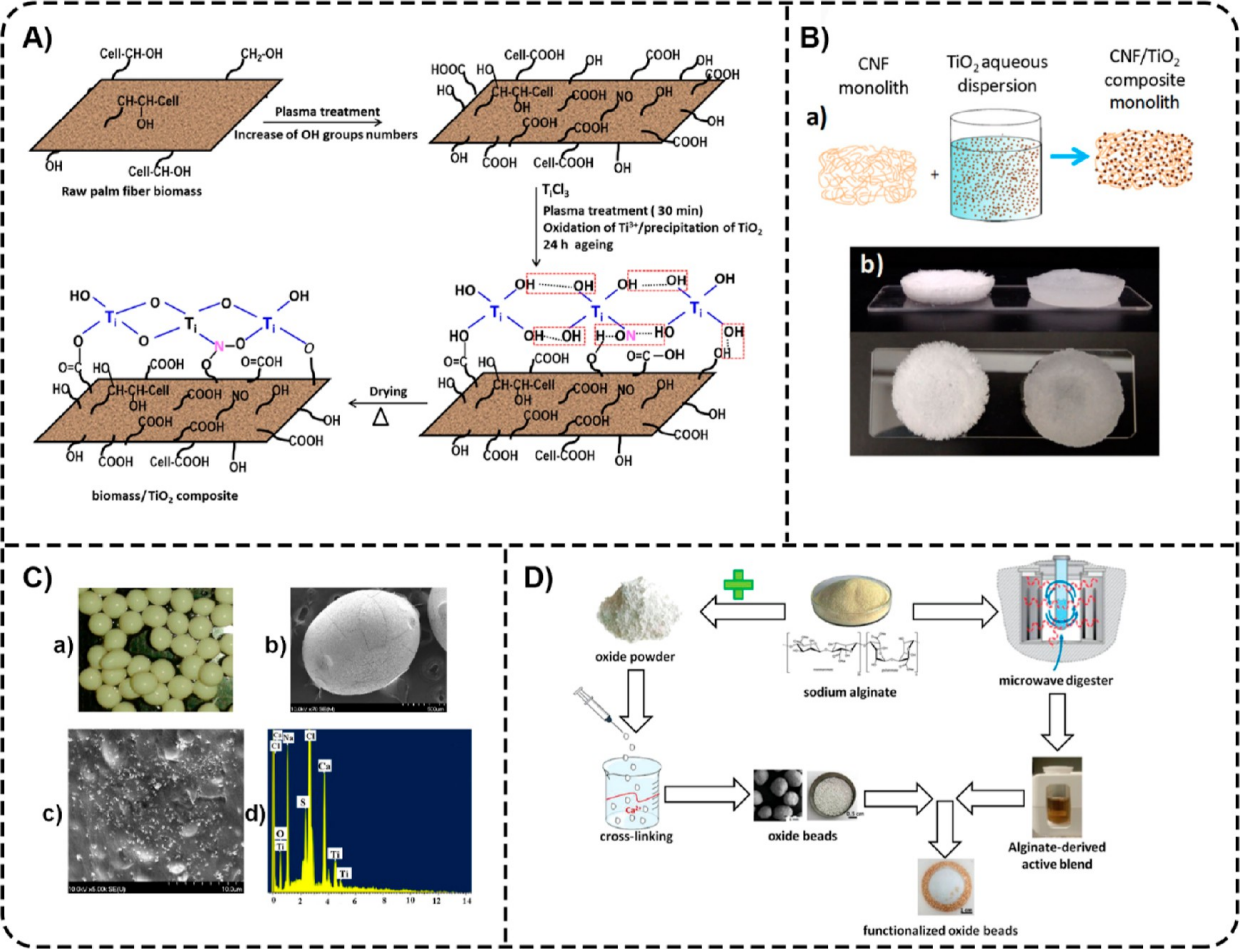
(A) The proposed mechanism of biomass/TiO_2_ composite synthesis. Adapted with permission from the ref 83 Copyright 2023 Springer Nature. (B) Schematic representation of the impregnation process used to fabricate CNF/TiO_2_ monoliths (a) and the physical appearances of the monoliths in dry (left) and wet (right) states (b). Adapted with permission from the ref 75 Copyright 2018 American Chemical Society. (C) Photograph of TIAB (a), SEM images of TIAB at different magnifications (b,c), and EDXA spectra of a single TIAB comprised of 4 wt % alginate beads and 2 wt % TiO_2_ NPs (d). Reprinted from the ref 84 Copyright (2015), with permission from Elsevier. (D) Scheme illustrating the preparation of oxide beads and functionalized oxide beads using sodium alginate as both a gelling agent and a precursor for a complex active blend containing carbon-based NPs. Reproduced with permission from the ref 86 Copyright 2020 John Wiley & Sons, Inc.

Some authors have developed alginate-based/TiO_2_ composites to remove pharmaceuticals in water treatment applications. A study by Sarkar et al. investigated the degradation of IBP using heterogeneous photocatalysis with alginate-supported catalyst beads (TIAB).^84^ The calcium alginate beads (Figure 6C) were synthesized by an ionotropic gelation method. An 85% IBP removal was achieved in continuous mode in a packed-bed photoreactor at a residence time of 20 min (at 25 °C and pH 7, with a substrate-to-catalyst ratio of 0.04). In another work, Vassalini et al. prepared spheroidal alginate/TiO_2_ (Alg/TiO_2_) oxide macrobeads with an average diameter of 0.9 ± 0.1 mm through ionotropic gelation and Alg/TiO_2_ oxide macrobeads functionalized with an alginate-derived active blend (Figure 6D).^86^ The authors studied the decontamination efficiency of these beads toward DCF and TCN. The functionalization of Alg/TiO_2_ oxide macrobeads exhibited positive effects for positively charged TCN and detrimental effects in the case of negatively charged DCF, with an increase in adsorption (+9%) and reduction of UV irradiation time (−120 min) for TCN (9.5 × 10^–5^ M) and a decrease in adsorption (−6%) and increase of UV irradiation time (+30 min) for DCF (1 × 10^–4^ M) observed. This study revealed the complete degradation of DCF and TCN under UV light over 60 and 240 min, respectively.

#### 6.1.4 Other Pharmaceutical Removal

Other types of pharmaceuticals, such as antiseptics, cardiovascular drugs, and anticonvulsant drugs, may be effectively removed from water by using natural polymer-based/TiO_2_ composites. For example, Sarkar et al. studied the degradation of aqueous solutions of CHD, ATL, and CBZ by heterogeneous photocatalysis with TIAB.^84^ In this study, 79% of CHD was removed by an adsorption process on TIAB after 1 h of contact. Moreover, 99% of CHD could be degraded by adsorption-photocatalysis within 2 h. In continuous mode, the percentage of CHD removed using a packed-bed photoreactor with TIAB as the packing material was 55% at a residence time of 60 min (at 30 °C and pH 10.5, with a substrate-to-catalyst ratio of 2.5). At the same time, ATL and CBZ removal percentages were 58% and 80% at a residence time of 60 and 40 min, respectively (at 25 °C and pH of 7 with a substrate-to-catalyst ratio of 0.04). The authors concluded that although TIAB showed lower degradation efficiency than TiO_2_ suspension, the catalyst’s recycling and reuse could make the water treatment process more cost-effective.

Reported studies have demonstrated the use of various natural polymer/TiO_2_ composites that efficiently eliminate a broad spectrum of pharmaceuticals from water, including antibiotics, NSAIDs, and other compounds. Furthermore, these studies have demonstrated various composite forms (membranes, fibers, beads, powders, etc.), different irradiation types (UV, visible, and sunlight), and varying treatment durations.

### 6.2 Synthetic Polymer/TiO_2_ Composites

#### 6.2.1 Synthetic Polymers

Despite the numerous advantages of natural polymers, their disadvantages limit their use; thus, synthetic polymers have attracted attention for water purification applications.^19,194^ Synthetic polymers are produced in laboratories and are typically derived from petroleum oil. The most common synthetic polymers are polyvinylpyrrolidone (PVP), poly­(ethylene glycol) (PEG), poly­(vinylidene fluoride) (PVDF), poly­(ethylene) (PE), poly­(styrene) (PS), poly­(ethersulfone) (PES), poly­(aniline) (PANI), poly­(methyl methacrylate) (PMMA), poly­(urethane) (PU), poly­(tetrafluoroethylene) (PTFE), poly­(vinyl chloride) (PVC), and poly­(vinyl alcohol) (PVA). Synthetic polymers have been utilized as materials for various applications, including energy,^195^ biomedical,^196^ food,^197,198^ water treatment,^199^ and others.^200^

#### 6.2.2 Antibiotic Removal

Synthetic polymers have been widely investigated for the removal of antibiotics (Table 5). For example, Malesic-Eleftheriadou et al. used recycled poly­(ethyleneterephthalate) (PET) to prepare PET-TiO_2_ composite films with different TiO_2_ contents (10%, 30%, and 47%) via the phase inversion method.^40^ The photocatalytic activities of the PET-TiO_2_ films were studied under simulated solar irradiation for the degradation of a mixture of antibiotics (INH, MNZ, SDZ, SMZ, TMP, NOR, MOX, and LIN). The mixture of antibiotics (1 mg L^–1^ for each tested compound) was nearly eliminated in 360 min, wherein LIN, MOX, INH, MNZ, and NOR were almost completely degraded within the first 2 h of irradiation, while the degradation efficiencies of TMP, SDZ, and SMZ were 90%, 93%, and 98%, respectively, after a 6 h treatment. In another work, Zhou et al. synthesized a novel nanocomposite based on poly­(butylene 2,5-furandicarboxylate) (PBF–TiO_2_) that was polymerized in situ using a two-step esterification and condensation procedure (Figure 7A).^41^ Photocatalytic degradation of six common antibiotics (MOX, NOR, SDZ, SMZ, TMP, and MNZ) was investigated under simulated sunlight. The results revealed that the degradation efficiencies of all antibiotics (5 mg L^–1^) were more than 75% within 210 min. At the same time, the values for SDZ, SMZ, and MNZ were >80% within 60 min, with MNZ showing the highest degradation efficiency. Meanwhile, Zhang et al. successfully formed oxygen- or nitrogen-linked heptazine-based polymer nanocomposites (TiO_2_/ONLH) through a simple hydrothermal method (Figure 7B) and utilized them to degrade fluoroquinolone (ENR, OFL, and CIP) and sulfonamide (SDZ, SMZ, and SMR) antibiotics under natural sunlight.^51^ Fluoroquinolones, containing *N*-piperazinyl were easily degraded. TiO_2_/ONLH nanocomposites (0.4 g L^–1^) exhibited superior performance toward the degradation of all the antibiotics (8 mg L^–1^) in 30 min, wherein more than 90% of ENR, OFL, and CIP, and more than 80% of SDZ, SMX, and SMR, were removed.

**Table 5:** Synthetic Polymer/TiO_2_ Composites for Antibiotic Removal

composite	target pharmaceuticals	composite form	irradiation	reusability	reference
antibiotics					
poly(ethyleneterephthalate)/TiO_2_ (PET-TiO_2_)	LIN, MOX, NOR, SMZ, SDZ, MNZ, TMP, INH	film	simulated sunlight	5 cycles	^40^
poly(butylene 2,5-furandicarboxylate)/TiO_2_ (PBF–TiO_2_)	MOX, MNZ, NOR, SMZ, SDZ, TMP	film	simulated sunlight	3 cycles for NOR and MOX	^41^
heptazine-base polymer/TiO_2_ (TiO_2_/ONLH)	CIP, ENR, OFL, SMZ, SDZ, SMR	powder	direct sunlight	N/A	^51^
poly(vinylidene fluoride)/TiO_2_ (NF TiO_2_@PVDF)	CIP	membrane	UV light (254 nm)	3 cycles	^52^
poly(vinylidene fluoride)/poly(vinylpyrrolidone)/TiO_2_ (PP/TiO_2_)	CIP	beads	UV light (367 nm)	30 cycles	^53^
poly(vinylidene fluoride-*co*-hexafluoropropylene)/TiO_2_ (TiO_2_/PVDF-HFP)	NOR	membrane	UV light (385 nm), visible light	N/A	^59^
poly(vinylidene difluoride)/*N*-doped TiO_2_ (NTiO_2_–PVDF)	SMZ	membrane	direct sunlight	N/A	^61^
poly(vinylidene fluoride)/conjugatedmicroporous polymer-acetylcysteine/TiO_2_ (PVDF-CMPs–HS–S/TiO_2_)	TC	membrane	visible light	4 cycles	^63^
conjugated microporous polymer/TiO_2_ (CMP/TiO_2_)	CIP, TC	powder	visible light	N/A	^54^
poly(ethersulfone)/trimesoyl chloride/diethylenetriamine/TiO_2_ (MTCN)	SMZ, TMP	membrane	N/A	N/A	^42^
poly(aniline)/TiO_2_ (TiO_2_/PANI)	MNZ	powder	UV light (365 nm), visible light	6 cycles	^46^
poly(aniline)/TiO_2_ (TiO_2_@PANI)	CIP	powder	UV light (290 nm)	5 cycles	^55^
poly(urethane)/TiO_2_ (TiO_2_@PU)	MNZ	foam	UV light (254 nm)	5 cycles	^47^
poly(urethane)/TiO_2_ (TiO_2_–PU)	TC	foam	simulated sunlight	5 cycles	^67^
poly(methylsilsesquioxane)/C-doped TiO_2_ (C–TiO_2_–PMSQ)	TC	aerogel	visible light	7 cycles	^71^

**Figure 7:**
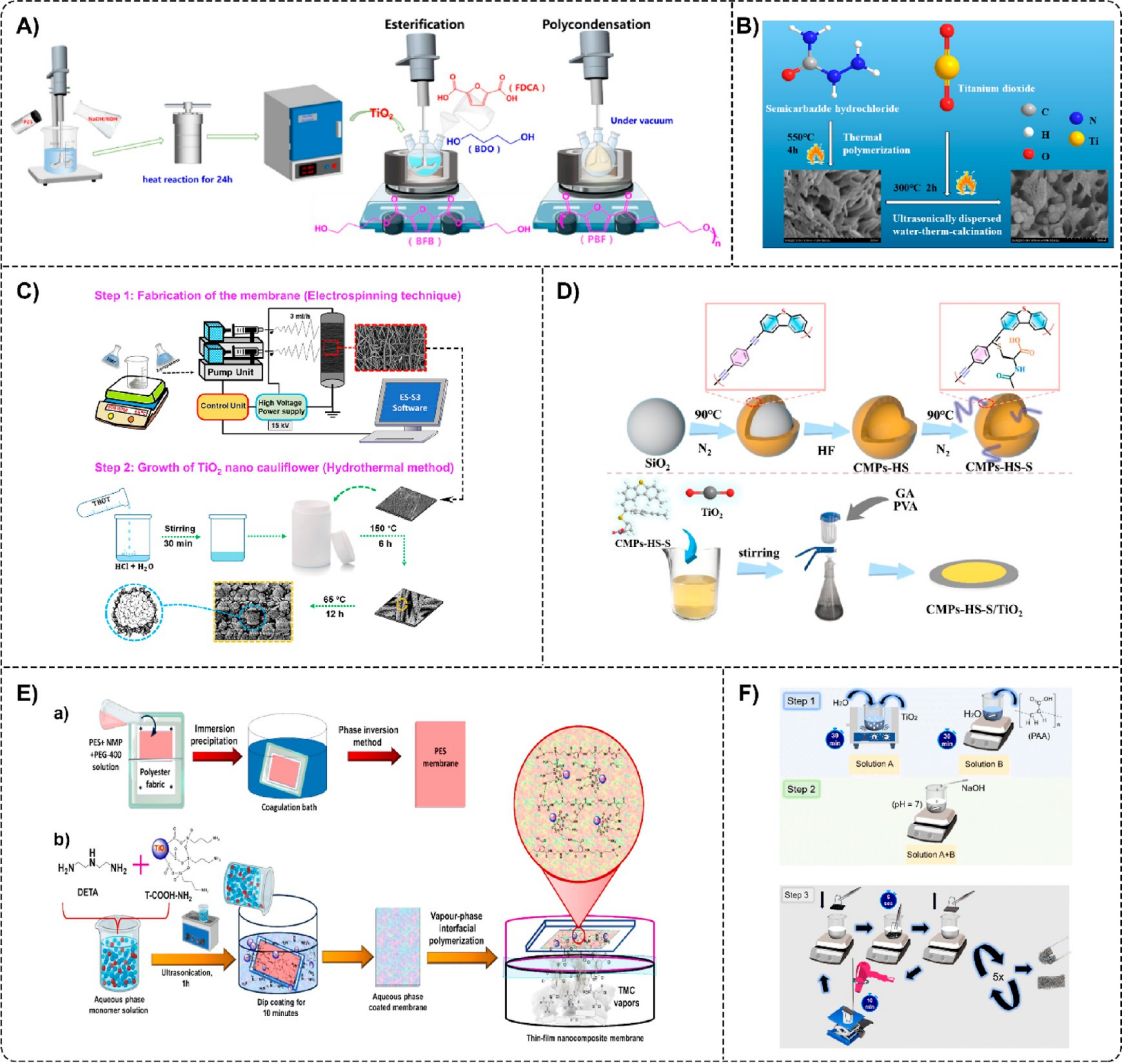
(A) Synthesis route of PBF composites. FDCA—2,5-furandicarboxylic acid, BDO—butanediol, BFB—(bis­(hydroxybutyl)-2,5-furan dicarboxylate. Reprinted from the ref 41 Copyright (2024), with permission from Elsevier. (B) Scheme for the fabrication process of TiO_2_/ONLH nanocomposites via hydrothermal calcination. Reprinted from the ref 51 Copyright (2021), with permission from Elsevier. (C) Schematic illustrating the fabrication of NF TiO_2_@PVDF nanocauliflower-like membranes. TBOT—titanium­(IV) butoxide. Reprinted with permission from the ref 52 Copyright 2023 American Chemical Society. (D) Flowchart illustrating the route for CMPs–HS–S/TiO_2_ composite preparation. GA—glutaraldehyde. Reprinted from the ref 63 Copyright (2025), with permission from Elsevier. (E) Schematic illustration of the fabrication process for the preparation of support PES membranes via the phase inversion method (a) and the formation of thin-film nanocomposite (TFN) membranes through the vapor-phase interfacial polymerization (VP-IP) technique (b). NMP—*N*-methyl pyrrolidone, PEG—poly­(ethylene glycol), DETA—diethylenetriamine, TMC—trimesoyl chloride. Reprinted from the ref 42 Copyright (2025), with permission from Elsevier. (F) Scheme for the fabrication of TiO_2_–PU foams. Steps 1 and 2 depict the preparation of dip-coating solutions, A and B, and A + B, for the impregnation of PU foams with TiO_2_. PAA—poly­(acrylic acid). Step 3 illustrates the dip-coating and drying processes for producing TiO_2_–PU foams. Reproduced from the ref 67 with permission from the Royal Society of Chemistry.

Among the synthetic polymers used in water treatment, PVDF has been extensively explored due to its chemical and UV resistance, thermal stability, mechanical strength, and low fouling. In one study, Ahmed and Dhar Purkayastha fabricated NF TiO_2_@PVDF membranes by preparing NF PVDF using an electrospinning process, followed by the growth of TiO_2_ nanocauliflower structures on its surface using a facile and cost-effective hydrothermal approach (Figure 7C).^52^ The degradation efficiency for CIF (10 mg L^–1^) reached 93% after 150 min of UV irradiation with the NF TiO_2_@PVDF membranes.

In another study, Raikar et al. prepared TiO_2_-immobilized PVDF (PP/TiO_2_) spongy beads by simple phase inversion of a mixture of TiO_2_ (1% w/v), PVDF (13% w/v), and PVP (0.7% w/v) for the degradation of CIP under UVA LED irradiation.^53^ The adsorptive removal of CIP (1 mg L^–1^) by PP/TiO_2_ beads was less than 45% after 60 min of dark incubation, while almost complete degradation was observed after 60 min under UV light. Moreover, CIP did not show antibacterial activity against *B. subtilis* and *E. coli* after 60 min of treatment, suggesting that CIP was degraded. The photodegradation of another quinolone antibiotic, NOR, under ultraviolet and visible radiation was investigated by Salazar et al. using solvent-cast and electrospun membranes of poly­(vinylidenefluoride-*co*-hexafluoropropylene) (PVDF-HFP) prepared with different TiO_2_ concentrations (TiO_2_/PVDF-HFP).^59^ The solvent-cast composite membranes, containing 3, 5, and 10 wt % of TiO_2_, showed degradation efficiencies of 33%, 35%, and 45%, respectively, after 90 min of UV irradiation. Under the same conditions, the electrospun composite membranes exhibited similar degradation efficiencies of 38%, 40%, and 45%, respectively. In contrast, less than 10% of NOR was degraded with all types of TiO_2_/PVDF-HFP membranes under visible radiation. Another PVDF-based composite system was developed by Nelson et al. to study the combined effect of sodium chloride concentration and pH on the photodegradation of SMZ under visible light.^61^ The novel nitrogen-doped TiO_2_ PVDF (NTiO_2_–PVDF) membranes showed optimum SMZ degradation of 76.5% in a solution of sodium chloride at a concentration of 7.8 g L^–1^ and pH 4.6, as predicted using the Response Surface methodology based on central composite design. More recently, Hasi et al. used PVDF membrane as a support material for conjugated microporous polymer (CMP) hollow spheres (CMPs-HS), which they modified with acetylcysteine (CMPs–HS–S) and compounded with TiO_2_ (CMPs–HS–S/TiO_2_) (Figure 7D), for efficient photocatalytic degradation of TC.^63^ Under visible light, the degradation efficiency of TC (10 mg L^–1^) reached 90% within 180 min. Additionally, when there was no TC present in the solution, the photocatalytic antibacterial activity of PVDF-CMPs–HS–S/TiO_2_ composites against *E. coli* and *S. aureus* was 89% and 99.99%, respectively. Similarly, Wu et al. employed a CMP/TiO_2_ composite system, synthesized by blending in situ polymerized CMPs with TiO_2_ under solvothermal conditions, for the degradation of CIP and TC under visible light.^54^ After 30 min, the degradation rates of CIP and TC (20.0 mg L^–1^) with CMP/TiO_2_ nanocomposites (10 mg) were 98.9% and 98.8%, respectively. Meanwhile, Yadav et al. designed TFN membranes for removing SMZ and TMP from water.^42^ The TFN membranes were fabricated by a vapor-phase interfacial polymerization method on PES support using an aqueous phase monomer of diethylenetriamine, incorporated with carboxylate-functionalized TiO_2_ (T-COOH) as well as amine-functionalized T-COOH nanofillers (T–COOH–NH_2_), and using an organic monomer phase of trimesoyl chloride vapors (Figure 7E). The highest degradation rates achieved were 99.26 ± 0.3% and 99.04 ± 0.5% for SMZ and TMP, respectively, and with 0.25 wt % of T–COOH–NH_2_ NPs incorporated in the TFN membrane (MTCN/0.25). Additionally, this study demonstrated that amine-functionalized T-COOH in TFN membranes improved their antifouling and antibacterial activities.

PANI is another synthetic polymer that is widely used in water purification. In one study, Asgari et al. prepared TiO_2_/PANI nanocomposites by in situ polymerization of aniline on TiO_2_ NPs and studied their photocatalytic activity for the degradation of MNZ in aqueous solution under UV and visible light irradiation.^46^ The maximum MNZ degradation efficiencies were found to be 98% and 96% after 120 min of UV and visible light irradiation, respectively, and with conditions at 1 g L^–1^ nanocomposite, 10 mg L^–1^ MNZ, and pH 7.0. Notably, the photocatalytic activity of TiO_2_/PANI nanocomposites under UV and visible light irradiation was higher than that of TiO_2_ NPs. By a similar approach, Prasetyo et al. synthesized TiO_2_@PANI composites via in situ polymerization of aniline monomer for attachment to the TiO_2_ surface in a chilled-alcohol jacketed reactor, followed by pyrolysis to enhance pore properties.^55^ The complete degradation of CIP (10 mg L^–1^) with TiO_2_@PANI (90%, 400 °C) was achieved in 240 min under UV light exposure.

Some authors investigated PU-based composites for the removal of antibiotics. For example, Sanches-Simões et al. used UV and UV/H_2_O_2_/O_3_ heterogeneous catalysis on TiO_2_ NPs functionalized in a highly porous PU foam (TiO_2_@PU) to estimate the degradation efficiency of MNZ.^47^ The results of this study revealed that UV/TiO_2_@PU was less effective, with 78% of MNZ degradation after 30 min of treatment compared to UV/H_2_O_2_/O_3_/TiO_2_@PU, which showed >97% degradation after 10 min of treatment. Meanwhile, Matias et al. synthesized TiO_2_ nanopowders using a fast microwave method, followed by their incorporation into PU foams via a simple dip-coating technique, to fabricate TiO_2_–PU composites (Figure 7F).^67^ The photocatalytic experiment showed around 80% TC degradation in the presence of the TiO_2_–PU foam after 180 min of simulated solar light irradiation. At the same time, Xu et al. proposed a highly porous carbon-doped TiO_2_-polymethylsilsesquioxane (C–TiO_2_–PMSQ) aerogel for the degradation of TC.^71^ The high surface area (747 g cm^–3^) C–TiO_2_–PMSQ aerogel removed 91% of TC (10 mg L^–1^) within 180 min under visible light, but due to the intrinsic hydrophobicity of PMSQ, an isopropyl alcohol/water cosolvent was needed for the removal. To overcome this limitation, the authors heat-treated the aerogel at 400 °C, decreasing its surface area to 618 g cm^–3^ and thus increasing its hydrophobicity, which in turn increased the removal efficiency of TC to 98% under aqueous conditions.

#### 6.2.3 Analgesic/Anti-Inflammatory Drug Removal

Synthetic polymer-based TiO_2_ composites for the removal of analgesic/anti-inflammatory drugs from water are summarized in Table 6. In one study, Eslami et al. used the polycarbonate substrate and nitrogen/sulfur-doped TiO_2_ (NS–TiO_2_) NPs to produce NS-TiO_2_@PC nanocomposites by a simple deposition method and explored the photocatalytic activity of the NS-TiO_2_@PC through degradation of IBP and NPX in a rectangular photocatalytic reactor (Figure 8A).^85^ At a contact time of 121 min, maximum degradation efficiencies of 80% and 98.1% under visible light irradiation (irradiation intensities of 8.36 mW/cm^2^ and 10 mW/cm^2^) were reached for IBP and NPX (10 mg L^–1^), respectively, and these experimental values were close to those predicted using Design Expert 7.0.0 software. Another study also reported on NPX degradation. As mentioned in Section 6.2.2. Antibiotic Removal of this review, Zhang et al. synthesized TiO_2_/ONLH nanocomposites for the degradation of antibiotics under natural sunlight.^51^ In addition to antibiotics, the authors also investigated the photocatalytic degradation of analgesic/anti-inflammatory drugs, NPX, and DCF. The TiO_2_/ONLH nanocomposites (0.4 g L^–1^) exhibited excellent photocatalytic activity with more than 80% of NPX and DCF (8 mg L^–1^) degraded after 30 min of exposure to natural sunlight.

**Table 6:** Synthetic Polymer/TiO_2_ Composites for Analgesic/Anti-Inflammatory Drug Removal

composite	target pharmaceuticals	composite form	irradiation	reusability	reference
analgesics					
poly(carbonate)/N,S–TiO_2_ (NS–TiO_2_@PC)	IBP, NPX	film	visible light	N/A	^85^
heptazine-base polymer/TiO_2_ (TiO_2_/ONLH)	NPX, DCF	powder	direct sunlight	N/A	^51^
poly(sulfone)/TiO_2_ (T–PSU)	DCF	membrane	UV light	N/A	^80^
poly(vinylidene fluoride)/TiO_2_ (T–PVDF)					
poly(tetrafluoroethylene)/TiO_2_ (T–PTFE)					
molecularly imprinted polymer/TiO_2_ (MIP25)	DCF	particles	UV light (366 nm)	6 cycles	^78^
poly(pyrrole)/TiO_2_ (PPy-TiO_2_)	DCF	powder	simulated sunlight	5 cycles	^79^
poly(ethersulfone)/TiO_2_ (TiO_2_/PES)	DCF, IBP	membrane	UV light	N/A	^77^
Poly(vinylidene fluoride)/TiO_2_ (TiO_2_/PVDF)					
poly(ethersulfone)/TiO_2_ (TiO_2_–PES)	DCF	membrane	UV light	N/A	^82^
poly(ethersulfone)/TiO_2_ (TiO_2_@PES)	AAP	film	UV light (365 nm)	6 cycles	^72^
poly(vinyl acetate)/TiO_2_ (PVAc@TiO_2_)	AAP	film	simulated sunlight	N/A	^73^
poly(methyl methacrylate)/TiO_2_ (PMMA@TiO_2_)					
poly(styrene)/TiO_2_ (PS@TiO_2_)					

**Figure 8:**
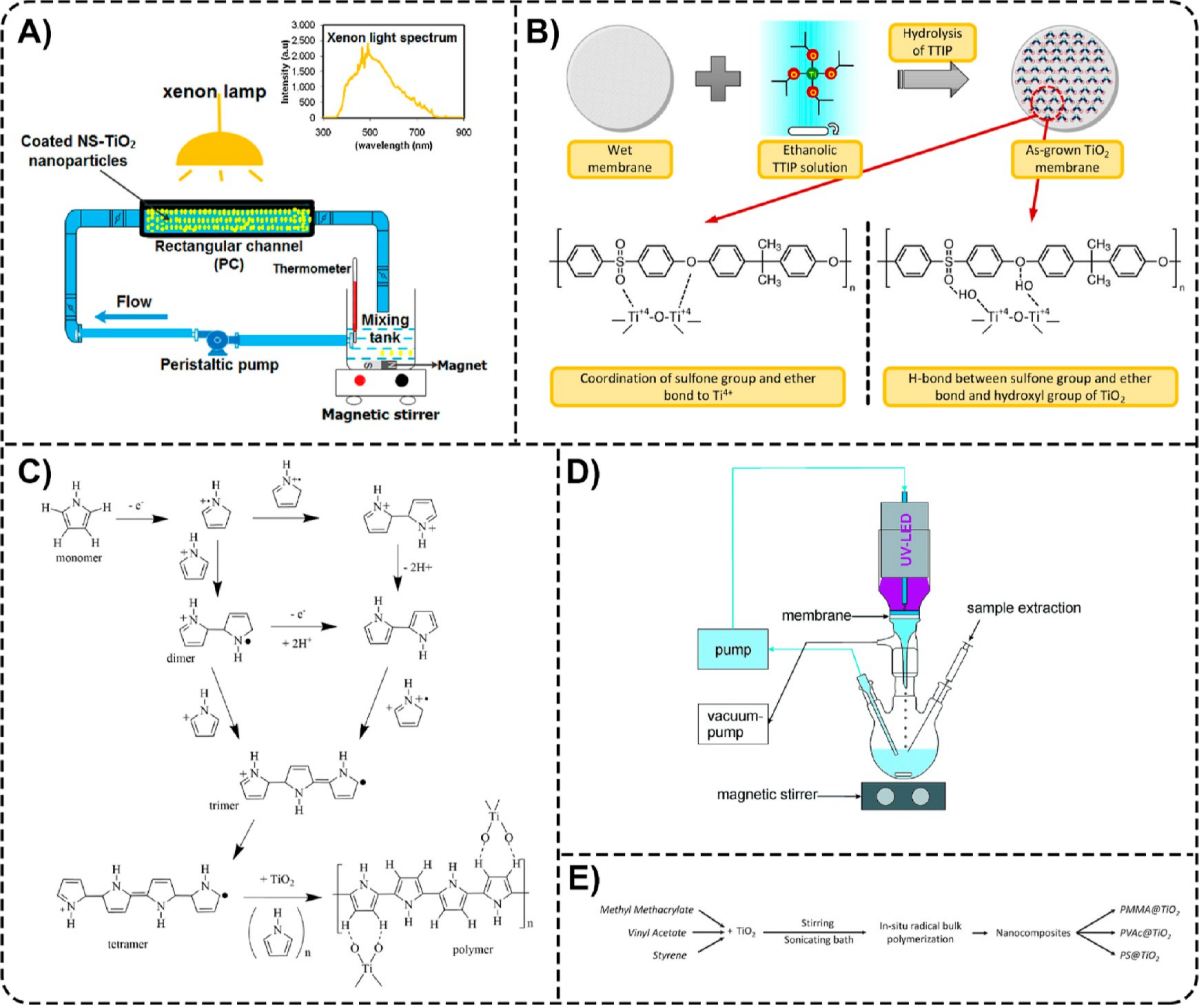
(A) Schematic illustration of the photocatalytic reactor containing NS-TiO_2_@PC nanocomposite and the experimental setup. Reprinted from the ref 85 Copyright (2020), with permission from Elsevier. (B) Schematic diagram for the fabrication of T–PSU membranes and the interaction of the PSU polymer matrix with TiO_2_. TTIP—titanium­(IV) isopropoxide. Reprinted from the ref 80 Copyright (2022), with permission from Elsevier. (C) Polymerization mechanism of pyrrole to poly­(pyrrole) in the presence of TiO_2_. Reprinted from the ref 79 Copyright (2020), with permission from Elsevier. (D) Schematic representation of the cross-flow setup used to evaluate the membrane’s photocatalytic performance in a flow-through mode. Reproduced from the ref 82 with permission from the Royal Society of Chemistry. (E) Simplified scheme of nanocomposite synthesis by in situ radical bulk polymerization of various polymer matrices. Reproduced with permission from the ref 73 Copyright 2023 John Wiley & Sons, Inc.

DCF is one of the most widely studied anti-inflammatory drugs in water treatment. In that regard, Dekkouche et al. grew TiO_2_ in situ on three commercial membranes (PSU, PVDF, and PTFE) to obtain composite membranes (T–PSU, T–PVDF, and T–PTFE) for the simultaneous removal of DCF and EE2 from water by adsorption and UV–LED photocatalysis (Figure 8B).^80^ After 24 h of UV–LED irradiation and continuous recirculation, all composite membranes achieved removal efficiencies greater than 90% for both DCF and EE2 (1.01 μmol L^–1^), with EE2 always preferentially adsorbed due to electrostatic repulsions between DCF and the membrane surfaces. Moreover, the stability of TiO_2_ on the membranes was tested by sonication and over several reaction cycles. The T–PVDF membrane presented the most stability after 4 days of UV–LED irradiation, while the T–PSU membrane, being the most active one, was damaged after the first adsorption-photocatalysis cycle. In another work, de Escobar et al. prepared molecularly imprinted polymers (MIPs) embedded with TiO_2_ (6.6% of total mass) (MIP25) for selective photocatalytic degradation of DCF.^78^ The adsorption capacity toward DCF was 8.6 mg g^–1^ after 60 min of adsorption experiments. After UV light irradiation for 300 min, the degradation of DCF (0.062 mmol L^–1^) with MIP25 reached 62.5%, which was 7-fold higher than that of its direct photolysis. The MIP25 photocatalyst demonstrated an excellent selectivity for DCF degradation over nontarget reference molecules, including PAR and fluoxetine (FLX). After 60 min of dark stage, the adsorptive removal of DCF, PAR, and FLX from the mixture was 32.6%, 4.9%, and 27.8%, respectively. After 120 min of the photodegradation stage, the degradation efficiencies of DCF, PAR, and FLX in the mixture were 48.7%, 17.1%, and 31.4%, respectively. At that time, Silvestri et al. proposed a PPy-based/TiO_2_ nanocomposite (PPy-TiO_2_), synthesized via polymerization using sulfuric acid (H_2_SO_4_) as the oxidizing agent (Figure 8C), for the removal of DCF under simulated solar light irradiation at environmental conditions.^79^ The results of this study showed that the PPy-TiO_2_ (1 g L^–1^) was able to remove more than 90% of DCF (10 mg L^–1^) in 60 min, which was higher than that of TiO_2_. Meanwhile, Fischer et al. prepared two hydrophilic membranes coated with crystallized TiO_2_ (TiO_2_/PES and TiO_2_/PVDF) and studied their photocatalytic activity toward the degradation of DCF and IBP.^77^ After 120 min under a UV-A sunlamp, 68% and 55% of DCF (25 mg L^–1^) were degraded by the TiO_2_/PES and TiO_2_/PVDF membranes, respectively, while the degradation efficiencies of IBP (100 mg L^–1^) were 45% and 50% for TiO_2_/PES and TiO_2_/PVDF, respectively, after 150 min of irradiation. In another study, Fischer et al. reported the combination of TiO_2_ nanotubes with a PES microfiltration membrane (TiO_2_–PES) to degrade DCF in a static and cross-flow experiment (Figure 8D).^82^ For the static setup, 94% of DCF (5 mg L^–1^) was degraded after 240 min, while its photolysis was slow and did not reach values higher than 30%. In a continuous way (cross-flow) with the TiO_2_–PES composite membrane, the degradation of DCF (5 mg L^–1^) was 42% after 4 days and 100% after 18 days of photocatalysis. The effect of fouling on the TiO_2_–PES composite membrane was observed after 10 days of the cross-flow experiment. In all, DCF toxicity was reduced by photocatalysis and photolysis, with photocatalysis having a greater effect.

Other authors fabricated TiO_2_@PES films with varying TiO_2_ contents (8, 11, 14, and 17 wt %) using the phase inversion method and studied their degradation of AAP under UV-A light in aqueous solution.^72^ The TiO_2_@PES film containing 14 wt % of TiO_2_ showed 51% degradation efficiency of AAP (10 mg L^–1^) for a film surface area of 160 cm^2^, while that increased to 80% at a film surface area of 320 cm^2^. However, increasing the TiO_2_ content to 17 wt % decreased degradation efficiency, and the authors primarily attributed this to TiO_2_ agglomeration and possible defective pore structures in the films. The photocatalytic degradation of AAP was also reported by Vento et al. The authors synthesized PMMA@TiO_2_, poly­(vinyl acetate) (PVAc)@TiO_2_, and PS@TiO_2_ nanocomposites via in situ radical bulk polymerization (Figure 8E), followed by the preparation of thin films of each nanocomposite.^73^ PMMA@TiO_2_ showed the highest degradation efficiency (around 40%) toward AAP (≈80 μM), followed by PVAc@TiO_2_ (25%), while PS@TiO_2_ exhibited the lowest efficiency (less than 5%) after 480 min under solar lamp irradiation.

#### 6.2.4 Other Pharmaceutical Removal

Synthetic polymer/TiO_2_ composites can also remove other pharmaceutical compounds (Table 7). For example, Yadav et al. fabricated TFN membranes to remove SMZ and TMP from water, as was described in Section 6.2.2. Antibiotic Removal of this review.^42^ Among the pharmaceuticals studied in this work, the degradation of the anticonvulsant drug, CBZ, was reported. The highest degradation rates were 65.63 ± 3.28%, 85.94 ± 4.29%, and 72.50 ± 3.62% with 0.5 wt % of T-COOH NPs (MTC/0.5), 0.25 wt % of T–COOH–NH_2_ NPs (MTCN/0.25), and 0.5 wt % of T–COOH–NH_2_ NPs (MTCN/0.5) incorporated in the TFN membranes, respectively. At that time, Fischer et al. developed another PES/TiO_2_ composite membrane to continuously remove CBZ (25 mg L^–1^) and achieved 60–70% CBZ degradation after 120 min under sunlamp irradiation.^88^ The PES/TiO_2_ membrane with evenly distributed TiO_2_ NPs (ultrasound-treated) had the highest photocatalytic activity. Another anticonvulsant drug, PGB, was effectively degraded using poly­(ethylene terephthalate)-based (PET/TiO_2_) composite beads.^89^ In this study, the authors used an antisolvent precipitation method to synthesize the PET/TiO_2_ beads with different contents of TiO_2_ (10, 20, and 30 wt %). Among these compositions, PET/TiO_2_ 30 wt % beads (1 g L^–1^) were found to be the most efficient, accomplishing almost complete degradation of PGB (10 mg L^–1^) within 6 h of xenon lamp irradiation (Figure 9A). PET-based nanocomposites (PET@TiO_2_) were also explored for the photocatalytic degradation of cytostatic drugs, including CPA, CYT, 5-FLU, and TAM.^91^ The complete degradation of TAM (1 mg L^–1^) was reached after 90 min under simulated solar light, while the degradation efficiencies toward CYT, 5-FLU, and CPA were 98%, 94%, and 90% within 6 h, respectively (Figure 9B).

**Table 7:** Synthetic Polymer/TiO_2_ Composites for Other Pharmaceutical Removal

composite	target pharmaceuticals	composite form	irradiation	reusability	reference
other pharmaceuticals					
poly(ethersulfone)/trimesoyl chloride/diethylenetriamine/TiO_2_ (MTCN)	CBZ	membrane	N/A	N/A	^42^
poly(ethersulfone)/TiO_2_ (PES/TiO_2_)	CBZ	membrane	simulated sunlight	N/A	^88^
poly(ethyleneterephthalate)/TiO_2_ (PET/TiO_2_)	PGB	beads	simulated sunlight	2 cycles	^89^
poly(ethylene terephthalate)/TiO_2_ (PET@TiO_2_)	TAM, CPA, CYT, 5-FLU	film	simulated sunlight	N/A	^91^
heptazine-base polymer/TiO_2_ (TiO_2_/ONLH)	PRL, ATL	powder	direct sunlight	4 cycles for PRL	^51^
poly(aniline)/TiO_2_ (TP)	PRL, APL	powder	UV light	N/A	^90^
poly(sulfone)/TiO_2_ (T–PSU)	EE2	membrane	UV light	N/A	^80^
poly(vinylidene fluoride)/TiO_2_ (T–PVDF)					
poly(tetrafluoroethylene)/TiO_2_ (T–PTFE)					
poly(vinylidene fluoride)/TiO_2_ (PVDF-TiO_2_)	CMT	nanofiber mat	UV light	10 cycles	^87^

**Figure 9:**
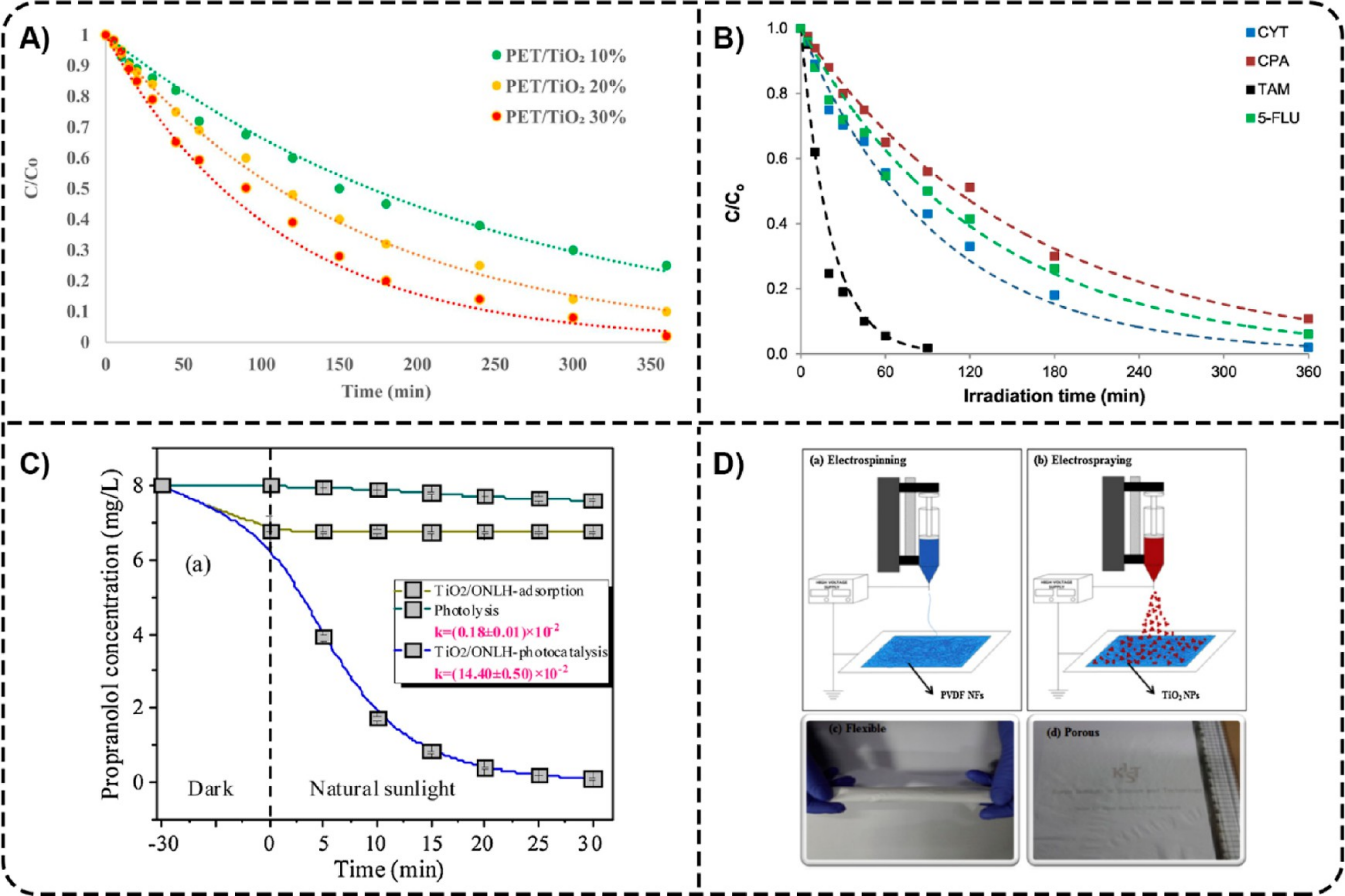
(A) Results from PGB photocatalytic degradation experiments using PET/TiO_2_ beads with different contents of TiO_2_. Reprinted from the ref 89 Copyright (2023), with permission from Elsevier. (B) Photocatalytic degradation of a cytostatic drug mixture using PET@TiO_2_ containing 10 wt % TiO_2_. Adapted from the ref 91 Copyright (2020), with permission from Elsevier. (C) PRL degradation via photolysis, TiO_2_/ONLH adsorption, and TiO_2_/ONLH photocatalysis under natural sunlight. Adapted from the ref 51 Copyright (2021), with permission from Elsevier. (D) Fabrication of PVDF-TiO_2_ photocatalysts by formation of PVDF nanofiber mats via electrospinning (a), deposition of TiO_2_ NPs on the PVDF nanofiber mats by electrospraying (b), and images showing the macrostructure and physical properties of the paper-like PVDF-TiO_2_ composite nanofibers (c,d). Reprinted from the ref 87 Copyright (2015), with permission from Elsevier.

Among pharmaceuticals detected in water, cardiovascular drugs, including ATL and PRL, have attracted attention in water treatment studies. For example, in the study mentioned above, Zhang et al. used TiO_2_/ONLH nanocomposites to degrade ATL and PRL under natural sunlight.^51^ The cardiovascular drugs having long aromatic side chains were more easily degraded, along with fluoroquinolones (Section 6.2.2. Antibiotic Removal). Therein, the TiO_2_/ONLH nanocomposites (0.4 g L^–1^) showed excellent photocatalytic performance toward the degradation of ATL and PRL (8 mg L^–1^), with more than 95% of the drugs degraded after 30 min of natural sunlight irradiation (Figure 9C). Šojić Merkulov et al. synthesized PANI-based nanocomposites (TP) with different molar TiO_2_:PANI ratios (TP-50, TP-100, and TP-150) and investigated their degradation efficiencies toward PRL and APL, an antidepressant drug.^90^ This study showed that 23% of PRL (50 μmol L^–1^) was removed using TP-50 and TP-100, while 15% was degraded using TP-150 within 60 min of UV radiation. In the case of APL, 45% and 32% were removed in the presence of TP-100 and TP-150, respectively, while TP-50 showed the lowest removal efficiency of 20%.

Some studies described the photocatalytic removal of hormones (EE2) and H2 blockers (CMT) from water. In this context, Dekkouche et al. investigated the photocatalytic activity of composite membranes (T–PSU, T–PVDF, and T–PTFE), as discussed in Section 6.2.3. Analgesic/Anti-Inflammatory Removal of this review, toward the removal of DCF and EE2.^80^ This study demonstrated high degradation efficiencies of EE2 (1.01 μmol L^–1^) of 96%, 94%, and 92%, along with high removal percentages of DCF, after 24 h of UV–LED irradiation for T–PSU, T–PVDF, and T–PTFE, respectively. In another work, Ramasundaram et al. created paper-like photocatalysts (PVDF-TiO_2_) with different TiO_2_ contents by electrospraying an *N*,*N*′-dimethylformamide dispersion of TiO_2_ NPs (10, 20, 40, and 60 mL) on PVDF nanofiber mats fabricated by electrospinning (Figure 9D).^87^ For the highest loading amount of TiO_2_, the complete degradation of CMT (10 μM) was accomplished within 40 min of UV irradiation.

Compared to natural polymer/TiO_2_ composites, the described studies reported a greater diversity in synthetic polymer/TiO_2_ composites for the removal of pharmaceuticals from water, efficiently eliminating a wide range of drug contaminants, including antibiotics, NSAIDs, and others. Furthermore, these studies demonstrated various approaches to composite formation, composite forms (membranes, fibers, films, beads, powders, and more), irradiation types (UV, visible, and sunlight), and treatment times.

#### 6.2.5 Assessment of the Toxicity of Synthetic Polymer/TiO_2_ Composites and Byproducts

Various compounds are formed during the photocatalytic degradation of pharmaceuticals. The toxicity of nanocomposites, as well as degradation products from their interactions with pharmaceuticals and drug residuals, needs to be addressed to avoid inadvertent secondary water contamination and harmful impacts on living organisms. Evaluating the toxicity and mitigating the ecological risks of nanocomposites and their photocatalytic byproducts are necessary to ensure their future safety.

Some authors estimated toxicity using a bioassay modeled with living aquatic organisms. For example, Farzadkia et al. assessed the toxicity of TiO_2_/PANI nanocomposites (1.0 g L^–1^) and the photocatalytic degradation products of MNZ (10 g L^–1^) by bioassay of freshwater crustacean *D. magna*, and the results showed moderate toxicity of TiO_2_/PANI nanocomposites.^201^ Photocatalytic degradation of MNZ by TiO_2_/PANI significantly reduced the effluent’s toxicity, suggesting that the degradation products were less harmful than the initial antibiotic compound. However, some residual toxicity remained even after complete MNZ degradation, indicating incomplete mineralization and the presence of intermediate MNZ degradation products. In another study, Matias et al. investigated the effects of TiO_2_–PU foams on the marine organism *A. salina* (brine shrimp) using an ecotoxicity assay.^67^ The results revealed that the TiO_2_–PU foams had no significant toxic effects and a low mortality rate (<10%) after 24 h under light irradiation; hence, they were considered safe for aquatic species.

Other authors used various cell lines to evaluate the toxicity of the photocatalytic byproducts. Fischer et al. studied the toxicity of intermediate products during photocatalytic degradation of DCF (25 mg L^–1^) with TiO_2_–PES membranes by examining the viability of yeast cells (*S. cerevisiae*) (Figure 10A).^82^ The initial DCF exhibited a high toxicity toward yeast cells (low cell viability), and using TiO_2_–PES membranes for the photocatalytic degradation of DCF led to increased cell viability. In static and cross-flow experiments, increasing the static irradiation time to 480 min and the cross-flow time to 21 days increased cell viability, demonstrating that the TiO_2_–PES membranes reduced the concentration of toxic byproducts over a longer time scale. In another work, Šojić Merkulov et al. used cell lines from rat hepatoma (H-4-II-E), mouse neuroblastoma (Neuro-2a), human colon adenocarcinoma (HT-29), and human fetal lung (MRC-5) to study the cytotoxicity of APL and its reaction mixtures with APL intermediates formed during photocatalytic degradation with TP-100.^90^ No significant cytotoxic effects on the growth of mammalian cell lines were observed after UV irradiation. The most sensitive cell line was HT-29, with toxicity levels ranging from 10 to 17%, and the highest cytotoxicity was observed in the APL reaction mixture after 60 min of irradiation (Figure 10B).

**Figure 10:**
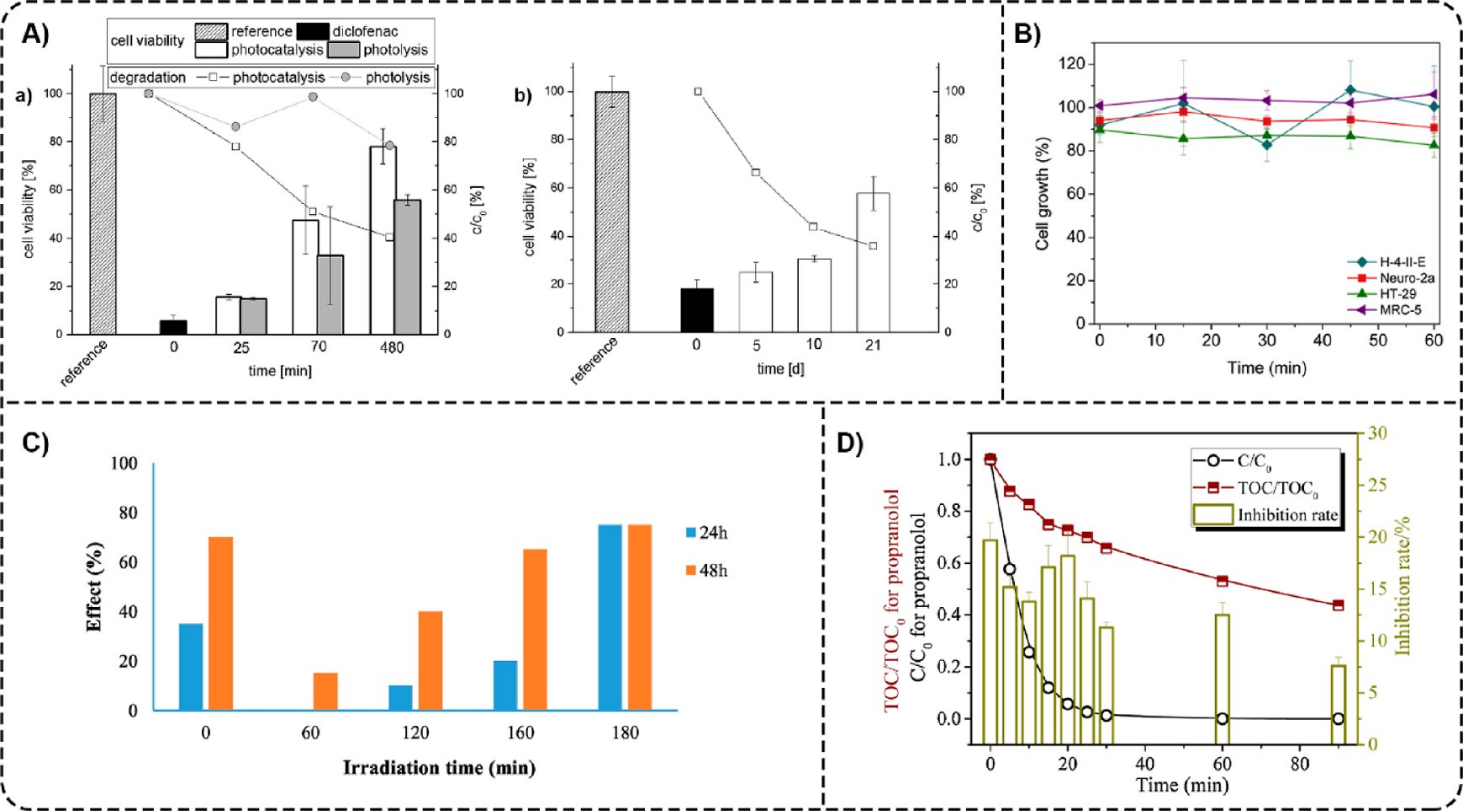
(A) The cell viability of yeast cells: untreated (reference), treated with DCF (25 mg L^–1^ at time = 0), and treated with DCF, which degraded by photocatalysis and photolysis over time in the static setup (a) and by photocatalysis over time in the cross-flow setup (b). DCF degradation profiles (photocatalytic and photolytic) over time are also presented as point–line graphs. Reproduced from the ref 82 with permission from the Royal Society of Chemistry. (B) Influence of reaction mixtures of APL and its byproducts, formed after treatment with TP-100 during different irradiation times, on the growth of select mammalian cell lines. Adapted from the ref 90 Copyright (2018), with permission from Elsevier. (C) Evolution of the toxicity of a cytostatic mixture on *D. magna* during photocatalytic treatment with PET@TiO_2_ containing 10 wt % TiO_2_. Reprinted from the ref 91 Copyright (2020), with permission from Elsevier. (D) Assessment of total organic carbon (TOC) removal (brown line, left *Y*-axis), PRL (8 mg L^–1^) degradation kinetics (black line, left *Y*-axis), and acute toxicity (*V. fischeri* exposure for 15 min, deep green column, right *Y*-axis) in using TiO_2_/ONLH nanocomposites under natural sunlight at pH 7.0 ± 0.2. Reprinted from the ref 51 Copyright (2021), with permission from Elsevier.

A study by Evgenidou et al. used the ECOSAR (Ecological Structure Activity Relationships) software for fish and algae to in silico predict the toxicity of PGB and its transformation products arising after photocatalytic degradation with PET/TiO_2_ beads.^89^ PGB and most of its generated transformation products were predicted to be nontoxic. However, amidation, as one of the transformation pathways, can result in the formation of potentially harmful and even toxic byproducts, which are susceptible to photocatalysis and can be degraded before the end of the treatment. In a related work, Evgenidou et al. evaluated the toxicity of a mixture of cytostatic drugs and their transformation products, formed during photocatalytic treatment with PET@TiO_2_ films, using a bioassay of *D. magna,* followed by ECOSAR analysis.^91^ The results showed that the toxicity of the mixture toward *D. magna* was reduced in the early stages of treatment but increased in the end (Figure 10C). In general, the toxicity of the mixture cannot be eliminated entirely, despite the decrease in the concentration of drugs in the mixture, and to reduce the ecotoxicity, prolonged irradiation times are needed. Zhang et al. also proposed prolonging the reaction time under natural sunlight to reduce the contaminant’s toxicity.^51^ The authors evaluated the toxicity of PRL and its mixture of transformation products produced by photocatalytic TiO_2_/ONLH (0.4 g L^–1^) composites via the inhibition of luminescent bacteria. An acute toxicity test revealed a gradual decrease in the PRL reaction solution’s toxicity as TOC levels decreased (Figure 10D). After 90 min, the percentage of bacterial inhibition was only 7.6%. According to the monomer toxicity evaluation, the TiO_2_/ONLH composites also reduced the formation of toxic transformation products. ECOSAR analysis indicated that the transformation products had lower acute and chronic toxicity than PRL.

These studies highlighted the importance of assessing the toxicity of polymer/TiO_2_ composites and byproducts generated during the photocatalytic degradation of contaminants to ensure their safe and sustainable production, thereby reducing potential adverse environmental impacts. Developing nontoxic polymer/TiO_2_ composites that completely degrade pharmaceuticals without any toxic effects of their transformation products is an immediate global challenge for the further development and application of water treatment technologies.

### 6.3 Hybrid Natural/Synthetic Polymer/TiO_2_ Composites

Conventional materials based solely on natural polymers are susceptible to breakage due to their reduced mechanical strength, stability, flexibility, and high degradability, which limits their practical applications. Therefore, these polymers can be blended or grafted with synthetic polymers to enhance the stability, hydrophilicity, and mechanical and physicochemical properties of polymer materials. Combining polymers that have complementary properties can yield an effective TiO_2_-supporting material for the photocatalytic degradation of pharmaceuticals.

#### 6.3.1 Antibiotic Removal

Hybrid natural/synthetic polymer/TiO_2_ composites used for antibiotic removal are summarized in Table 8. In one study, Pradhan et al. proposed an in situ one-pot approach, wherein TiO_2_ quantum dots (QDs) were synthesized with simultaneous grafting of methacrylic acid (MAc) on guar gum (GG) to fabricate hybrid composites (GG-*g*-PMAc-@-TiO_2_ QDs) (Figure 11A) for the degradation of CIP.^56^ The degradation efficiency of CIP (10 ppm) reached approximately 94% within 3 h of UV light irradiation. Furthermore, the degradation efficiencies were approximately 55% and 61% under visible and solar light, respectively.

**Table 8:** Hybrid Natural/Synthetic Polymer/TiO_2_ Composites for Antibiotic Removal

composite	target pharmaceuticals	composite form	irradiation	reusability	reference
antibiotics					
guar gum-methacrylic acid/TiO_2_ quantum dots (GG-*g*-PMAc-@-TiO_2_ QDs)	CIP	powder	UV light	5 cycles	^56^
alginate/poly(vinylpyrrolidone)/TiO_2_ (TiO_2_–AP)	ENR, OTC	beads	UV light	6 cycles	^57^
poly(aniline)/cellulose/TiO_2_ (PANI/TiO_2_/NCF)	TC	fiber disc	simulated sunlight	N/A	^68^
poly(vinyl alcohol)/chitosan/TiO_2_ (PVA–CS–TiO_2_)	MNZ, TC, CEF	beads	UV light (254 nm)	3 cycles	^39^
poly(vinyl alcohol)/chitosan/TiO_2_ (PVA–CS–TiO_2_)	MNZ	beads	UV light (254 nm)	15 cycles	^45^

**Figure 11:**
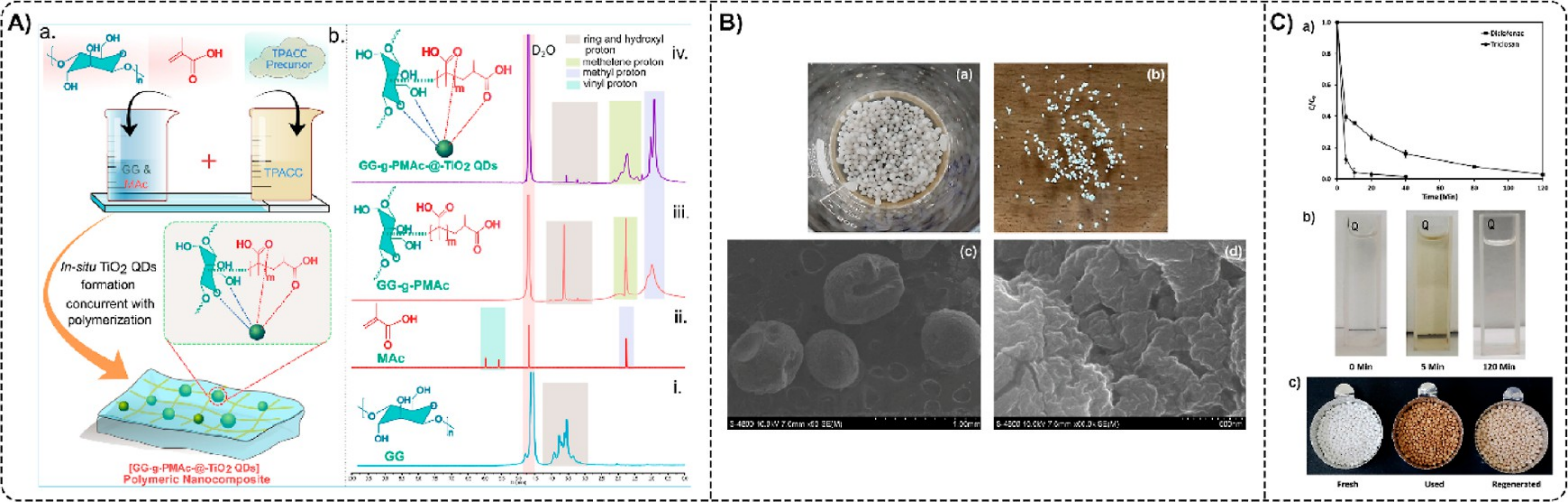
(A) Schematic illustration of the GG-*g*-PMAc-@-TiO_2_ QD synthesis process (a) and proton nuclear magnetic resonance (^1^H NMR) spectra (b) of GG (i), MAc (ii), GG-*g*-PMAc (iii), and GG-*g*-PMAc-@-TiO_2_ QDs (iv). TPACC—titanium peroxo ammonium carbonate complex. Reprinted from the ref 56 Copyright (2025), with permission from Elsevier. (B) Photographs of synthesized TiO_2_–AP1–0.2 hydrogel beads in wet (a) and dry (b) states. SEM images of the TiO_2_–AP1–0.2 bead surface at two low (c) and high (d) magnifications. Reprinted with permission from the ref 57 Copyright 2024 Springer Nature. (C) Results from the photocatalytic degradation of pharmaceuticals using PAT-12 M: degradation kinetics of DCF and TCS (a), digital images of DCF samples collected at different time intervals, showing the formation and disappearance of colored byproducts (b), and digital images of PAT-12 M beads at various time points (c). Reprinted from the ref 81 Copyright (2020), with permission from Elsevier.

Nguyen et al. studied the photocatalytic degradation of another quinolone antibiotic, ENR, as well as a tetracycline antibiotic, OTC, using TiO_2_-loaded hydrogel beads (TiO_2_-AP) (Figure 11B), prepared by the sol–gel method of alginate cross-linked in CaCl_2_.^57^ The TiO_2_ hydrogel beads were synthesized in two ways: (1) sodium alginate was mixed with PVP and TiO_2_ (0.2, 0.4, and 0.6 g), followed by cross-linking (TiO_2_–AP1 composites) or (2) hydrogel beads were soaked in CaCl_2_ solution containing TiO_2_ (TiO_2_–AP2 composites). It was shown that TiO_2_–AP1 beads exhibited superior performance compared to TiO_2_–AP2 beads. Within 240 min of UV irradiation, the TiO_2_–AP1–0.4 composites exhibited the highest removal efficiencies for ENR and OTC (20 mg L^–1^) of 89.5% and 82.9%, respectively. This study revealed that the photocatalytic activity of TiO_2_–AP1 beads was slightly lower than that of TiO_2_ NPs; however, their separation and recovery abilities were significantly improved. Meanwhile, Liu et al. developed a facile solar water evaporator device based on natural lignocellulose fibers (NCF), PANI, and TiO_2_ and investigated the photocatalytic degradation of TC under simulated solar illumination.^68^ Therein, photothermal PANI nanofibers were synthesized on the surface of NCF, followed by the addition of TiO_2_ NPs to the polymerization reaction to form a hybrid PANI/TiO_2_/NCF composite disc. This device was then used to evaporate water for 10 h with TC solution (100 ppm) as the base solution (the liquid in the beaker). The results of this study showed that due to the synergistic effect between photothermal PANI and photocatalytic TiO_2_ NPs in the solar water evaporator, a water evaporation rate of 2.36 kg m^–2^ h^–1^ (under single sunlight irradiation) can be achieved, while efficiently degrading TC. The photocatalytic degradation of TC, as well as CEF and MNZ, was investigated by Neghi and Kumar using PVA–CS–TiO_2_ composite beads synthesized via the chemical precipitation of a PVA–CS–TiO_2_ blend in an alkali/solvent medium.^39^ The maximum removal percentages after 120 min under UV irradiation were 89.3%, 54.5%, and 99.9% for TC, CEF, and MNZ (0.1 mg L^–1^), respectively, and hydrophilic MNZ showed faster degradation than the hydrophobic TC and CEF. Under multicompound conditions, the degradation efficiencies decreased to approximately 70%, 33%, and 93% for TC, CEF, and MNZ (0.1 mg L^–1^), respectively. In another work, Neghi et al. used the same PVA–CS–TiO_2_ beads for the photocatalytic removal of MNZ in a batch reactor.^45^ The composites exhibited 100% MNZ (10 mg L^–1^) degradation within 120 min at a catalyst loading of 0.3 g L^–1^, implying that the removal of MNZ was due to the synergistic action of both adsorption and photocatalysis.

#### 6.3.2 Analgesic/Anti-Inflammatory Drug Removal

Several papers focus on the use of natural/synthetic polymer/TiO_2_ composites for the removal of analgesic and anti-inflammatory pharmaceuticals (Table 9). Mukherjee et al. developed five different PVA/PVP/gelatin polymeric membranes with immobilized TiO_2_ (PVA/PVP/G/TiO_2_) and cross-linked with various treatments, including heat, aldehyde, UV, freeze-drying, or UV-freeze-drying.^76^ The photocatalytic degradation of ASP under both UV and solar light irradiation was investigated. The freeze-dried PVA/PVP/G/TiO_2_ membranes showed a higher degradation efficiency under both solar and UV lights; however, they were not mechanically strong and became unstable after 6–7 h. Meanwhile, the UV-treated and UV-freeze-dried PVA/PVP/G/TiO_2_ membranes showed the lowest degradation efficiency toward ASP. In another study, Mehmood et al. reported a facile method for the preparation of poly­(sulfone)/alginate/TiO_2_ (PAT) composite beads with various ratios of polysulfone:alginate:TiO_2_.^81^ With surface modification, the PAT beads with a final w/v ratio of poly­(sulfone):alginate:TiO_2_ being 10:6:12 (PAT-12 M) showed 60% DCF (20 mg L^–1^) removal within 5 min and almost complete removal of DCF within 120 min of UV irradiation (Figure 11C).

**Table 9:** Hybrid Natural/Synthetic Polymer/TiO_2_ Composites for Analgesic/Anti-Inflammatory and Other Pharmaceutical Removal

composite	target pharmaceuticals	composite form	irradiation	reusability	reference
analgesics					
poly(vinyl alcohol)l/poly(vinylpyrrolidone)/gelatin/TiO_2_ (PVA/PVP/G/TiO_2_)	ASP	membrane	UV light, simulated sunlight	N/A	^76^
poly(sulfone)/alginate/TiO_2_ (PAT-12 M)	DCF	beads	UV light (254 nm)	N/A	^81^
other pharmaceuticals					
poly(sulfone)/alginate/TiO_2_ (PAT-12 M)	TCS	beads	UV light (254 nm)	N/A	^81^

#### 6.3.3 Other Pharmaceutical Removal

In addition to photodegradation of DCF, Mehmood et al. also studied the photocatalytic degradation of TCS using PAT-12 M composite beads.^81^ These beads demonstrated faster degradation of TCS (20 mg L^–1^) compared to DCF, with 87% TCS removal within 5 min and almost complete removal of TCS within 40 min of UV irradiation (Figure 11C).

The studies reported highlight a lower diversity in hybrid natural/synthetic polymer/TiO_2_ composites for pharmaceutical removal from water compared to the natural or synthetic polymer/TiO_2_ composites described in Section 6.1. Natural Polymer/TiO_2_ Composites and Section 6.2. Synthetic Polymer/TiO_2_ Composites of this review, respectively. Among the composite forms, beads were the most utilized among the hybrid natural/synthetic polymer/TiO_2_ composites reported in this review. Nevertheless, these composites have demonstrated high efficiency in pharmaceutical removal and may therefore attract more attention in future studies.

### 6.4 Reusability of the Polymer/TiO_2_ Composites

Following the principles of “cleaner technologies”,^202^ the reusability of polymer/TiO_2_ composites is a desirable characteristic for their practical application from both environmental and economic perspectives. The more regeneration cycles the composites can undergo while also maintaining high degradation efficiencies of the target pharmaceuticals, the more resource-conservative and cost-effective the water treatment process will be. Focusing on reusability is crucial to addressing environmental concerns and improving the economic viability of polymer/TiO_2_ composite materials. The reusability of polymer/TiO_2_ composites is presented in Tables 3–9.

#### 6.4.1 Reusability of the Natural Polymer/TiO_2_ Composites

Rinsing with water or other aqueous solutions is a simple, cost-effective, and energy-efficient approach to regenerating polymer/TiO_2_ composites for multiple reuse cycles. For example, Zou et al. washed the C-SLCNF-TNPs aerogels with deionized water and then freeze-dried them after each use cycle.^62^ The aerogels showed a slight decrease in removal efficiency from 94.9% to 89.5% for TC after five cycles, while maintaining their mechanical strength and geometric shape. In another study, Kanmaz et al. carried out reusability experiments by shaking a TC-loaded 80% TiO_2_@EC with 20 mL of 0.1 M NaOH solution at 140 rpm for 120 min^64^ After that, the desorbed adsorbent was reloaded with 20 mL of a 30 mg L^–1^ TC solution. The following desorption steps were performed in the same way as the initial desorption procedure. Figure 12A illustrates the reusability of 80% TiO_2_@EC with a 0.1 M NaOH eluent solution. While approximately 92% of TC removal was achieved after the first loading, it decreased to 72% after the fifth cycle. At the same time, TC recovery from the composite surface was 80% after the first cycle and 57% by the end of the fifth cycle. Meanwhile, Bergamonti et al. investigated the reusability of the TiO_2_/CS scaffolds after three 180 min cycles of UV irradiation each by washing in distilled water as a regeneration step.^48^ The TiO_2_/CS scaffolds, composed of five layers, at a molar ratio of AMX:TiO_2_ 1:100, degraded about 80% of the AMX after the third cycle. In the case of the three-layer scaffold, only 60% of AMX was degraded after the third cycle (Figure 12B). Ikhlef-Taguelmimt used a similar regeneration procedure for CS@TiO_2_ composite films and instead studied the films’ reusability over four successive cycles.^69^ The removal of TC decreased significantly after the second run, from 87% to 57%, and then decreased slightly in the third (54%) and fourth (52%) runs. Additionally, the CS@TiO_2_ film, used four times, was washed with distilled water and a 0.1 M NaOH solution after the first reuse cycle, resulting in an increased film degradation efficiency (58%).

**Figure 12:**
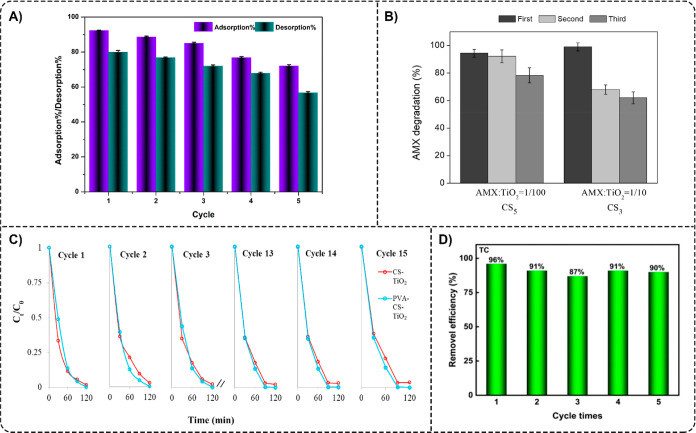
(A) Reusability of 80%TiO_2_@EC during TC adsorption. The dark violet columns represent the percentage of TC adsorbed, while the dark green columns indicate the recovery percentage of TC adsorbed on the composite surface. Reprinted from the ref 64 Copyright (2023), with permission from Elsevier. (B) Degradation efficiency of TiO_2_/CS_5_ and TiO_2_/CS_3_ scaffolds toward AMX after three photodegradation cycles. Reprinted from the ref 48 Copyright (2019), with permission from Elsevier. (C) Removal profiles of MNZ over 15 cycles using CS-TiO_2_ and PVA–CS–TiO_2_ composites. Reprinted from the ref 45 Copyright (2019), with permission from Elsevier. (D) TC photodegradation over multiple photocatalytic cycles with recycled β-CDP/TiO_2_ photocatalysts. Reprinted from the ref 65 Copyright (2024), with permission from Elsevier.

In another study, Farhadian et al. evaluated the reusability of NST/CS composites over four cycles of TC removal.^70^ At the end of each cycle, the composites were centrifuged, washed with water three times, dried at 70 °C overnight, recovered, and reused without further treatment. After the first cycle, the NST/CS composites exhibited a 25% reduction in TC degradation efficiency, while the degradation efficiency remained constant in subsequent runs. In another work, Dionmbete et al. studied the reusability of LCB/TiO_2_ composites over five cycles of DCF degradation under gliding arc discharge irradiation.^83^ After each cycle, the LCB/TiO_2_ composites were recovered by centrifugation, washed with distilled water, and dried at 105 °C. Removal efficiencies of the LCB/TiO_2_ composites toward DCF decreased incrementally on the first, second, third, fourth, and fifth cycles from 98%, 77%, 70%, 56%, and to 44%, respectively. Meanwhile, Jallouli et al. irradiated TiO_2_–CF films in a PAR solution and evaluated their degradation performance after five cycles of regeneration and reuse.^74^ For that, the films were washed with deionized water following irradiation and then replenished with fresh PAR solution for reexposure to irradiation. This procedure was repeated for several more cycles, and it was found that each cycle of film regeneration resulted in slightly reduced performance: after the first cycle, the degradation efficiency was 83%, while after five cycles it was about 80%, indicating the film could be reused several times for the removal of PAR from water. Sarkar et al. investigated the reusability of TIAB over five cycles in continuous operation using PBPR composites for the degradation of CHD.^84^ After each cycle, the catalyst was recovered by washing in mildly acidic water and used again, and the results showed a decrease in the degradation efficiency from 99% to 85% after five continuous reuse cycles.

Another approach for the recovery and recycling of polymer/TiO_2_ composites is the use of UV irradiation to degrade pharmaceutical compounds. Hence, Huang et al. regenerated TiO_2_@AMSF composites from a CIP solution by using UV irradiation at the end of each reuse cycle.^49^ The CIP removal ability of the composites over three running cycles, with each cycle lasting 7 h under natural light, was studied across two different forms of TiO_2_@AMSF, containing either one or three TiO_2_-gel layers. The maximum attenuation rate of CIP removal was less than 40% for TiO_2_@AMSF containing one layer and less than 20% for that containing three layers; therefore, the latter was more stable. In another study, Rizzi et al. showed that β-EPI-TiO_2_ nanocomposites could be regenerated through continuous irradiation with UV light and pulsed light for the photolysis of adsorbed SMZ.^60^

At that time, Rizzi et al. proposed various approaches to recover mAL/CH/TiO_2_ microbeads.^66^ The first approach involved 1 h of stirring for the microbeads that were previously loaded with TC in an aqueous solution of MgCl_2_ (2 M), resulting in almost 100% release of the adsorbed TC. In the second approach, the microbeads were exposed to UV irradiation for 2 h after the first cycle of adsorption, and nearly 100% of the adsorbed TC was degraded. The second adsorption cycle achieved a TC removal efficiency of approximately 80%. Furthermore, three consecutive adsorption cycles without UV exposure were performed, and the TC removal efficiency (∼50% per cycle) remained constant across all three cycles. Almost complete degradation of TC was achieved after 3 adsorption cycles with 4 h of UV irradiation. The final recovery strategy investigated employed UV irradiation and H_2_O_2_ washing after the adsorption cycle to facilitate complete TC degradation within 15 min.

The reusability of the composites was also studied without any regeneration procedure. For example, the excellent reusability of CS-TiO_2_ composites was demonstrated by repeating photocatalytic experiments (120 min) 15 times with the same composite material, which was retrieved (using a metallic tea strainer mesh) after each cycle.^45^ Analysis of transmission electron microscopy images of the CS-TiO_2_ composites revealed a slight loss in TiO_2_ content after 15 cycles of treatment, which might account for the decrease in MNZ removal from complete degradation to 97.9% at the 13th cycle in CS-TiO_2_ (Figure 12C). In another study, Zhang et al. evaluated the reusability of β-CDP/TiO_2_ composites for photocatalytic degradation of TC.^65^ It was shown that the removal efficiency remained above 87% after five cycles (Figure 12D), indicating that the β-CDP/TiO_2_ composites could be easily recovered. Meanwhile, Villanueva et al. reported K–TiO_2_ composites containing 10% w/w TiO_2_, which could be reused for TMP removal for at least four cycles without a significant decrease in removal efficiency.^43^

Most studies reported reusability of natural polymer/TiO_2_ composites for up to five cycles, except for the study by Neghi et al., which demonstrated reusability of CS-TiO_2_ composites for 15 cycles.^45^ Therefore, designing highly stable natural polymer/TiO_2_ composites while maintaining an adequate degradation efficiency toward pharmaceuticals is a critical consideration in scaling the production of composites for water remediation.

#### 6.4.2 Reusability of the Synthetic Polymer/TiO_2_ Composites

As with natural polymer/TiO_2_ composites, water rinsing is the primary technique for regenerating synthetic polymer/TiO_2_ composites for multiple reuse cycles. Hence, Malesic-Eleftheriadou et al. evaluated the photocatalytic stability of PET-10 wt %TiO_2_ composite films over five cycles of reuse by washing the films with deionized water following each photocatalytic cycle.^40^ The results showed that the composite films had high performance, even after five cycles. In another study, Matias et al. investigated the reusability of a TiO_2_–PU foam by conducting five consecutive cycles of TC degradation and TiO_2_–PU foam regeneration.^67^ To recover the composite, the functionalized TiO_2_–PU foam was washed with deionized water only before the following photocatalytic cycle. After the fifth cycle, the foam’s degradation efficiency decreased by approximately 20% from the initial 80% (Figure 13A).

**Figure 13:**
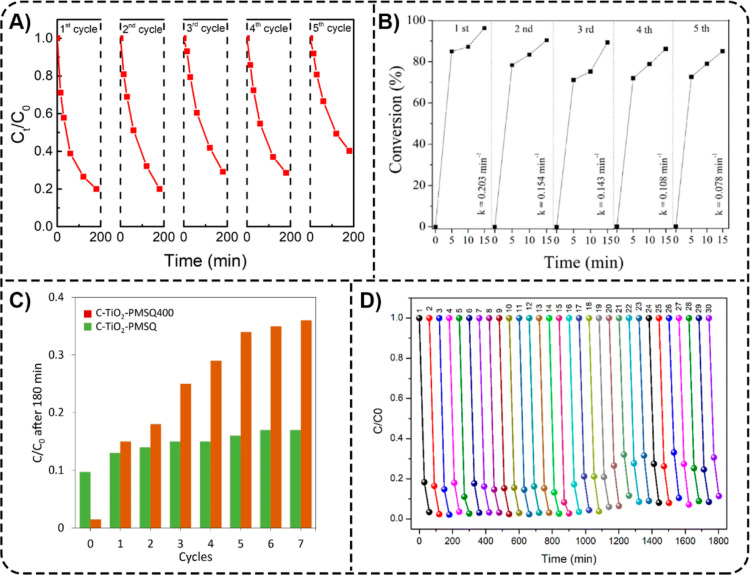
(A) Photocatalytic recycling performance of pretreated TiO_2_–PU foams over five consecutive 180 min cycles of simulated solar irradiation. Reproduced from the ref 67 with permission from the Royal Society of Chemistry. (B) Reusability of PPy-TiO_2_ composites across multiple DCF degradation cycles. Adapted from the ref 79 Copyright (2020), with permission from Elsevier. (C) Degradation stability of C–TiO_2_–PMSQ (green bars) and heat-treated C–TiO_2_–PMSQ400 (brown bars) aerogels after seven 180 min cycles of irradiation. Reprinted with permission from the ref 71 Copyright 2021 Springer Nature. (D) Results of reusability experiments with PP/TiO_2_ beads. Reproduced from the ref 53 with permission from the Royal Society of Chemistry.

Similarly, Silvestri et al. tested the reusability of PPy-TiO_2_ composites over five consecutive cycles; after each cycle, the composites were recovered by several washes with deionized water before the next cycle.^79^ The results demonstrated the ability of PPy-TiO_2_ to degrade DCF with an efficiency above 90% after the fifth cycle (Figure 13B). Meanwhile, Chijioke-Okere et al. studied the stability and reusability of photocatalytic TiO_2_@PES films during the degradation of AAP.^72^ The film was washed with distilled water after each degradation cycle, and the reusability study demonstrated a high stability of the TiO_2_@PES films with comparable degradation efficiencies across five cycles before a slight decrease in efficiency in the sixth run. In another study, Ramasundaram et al. examined the catalytic performance of PVDF–TiO_2_ hybrids in the degradation of CMT over 10 cycles.^87^ After each cycle, the composites were withdrawn from the treated water and washed with deionized water before being returned to the CMT solution for the next run. The results showed no noticeable reduction in photocatalytic activity after 10 cycles, suggesting good potential for the PVDF–TiO_2_ composites for recycling.

Another washing solution, a mixture of distilled water and isopropyl alcohol, was used by Xu et al. to investigate the photocatalytic performance stability of C–TiO_2_–PMSQ and C–TiO_2_–PMSQ400 composite aerogels during seven cycles of TC degradation (Figure 13C).^71^ After degradation, the aerogels were washed three times with a 50/50 (v/v %) distilled water/isopropyl alcohol mixture, followed by three washes with isopropyl alcohol, and then dried at 170 °C for 1 h before the next cycle of TC degradation. The reusability experiments showed that, compared to the initial cycles, degradation efficiencies decreased to 64% and 83% after the seventh regeneration cycle for the C–TiO_2_–PMSQ400 and C–TiO_2_–PMSQ aerogels, respectively.

Some authors used only the separation of composites from treated water to investigate their reusability. For example, Raikar et al. reported the excellent reusability of PP/TiO_2_ beads for the photocatalytic degradation of CIP and demonstrated that suspended PP/TiO_2_ beads could be easily recovered post-photocatalysis using a simple strainer.^53^ After 20 cycles, no reduction in the photocatalytic activity of the beads was observed, whereas after 30 cycles, the degradation efficiency was mostly preserved at 90% (Figure 13D), with no significant leaching of TiO_2_ into the water.

At that time, Asgari et al. conducted a reusability test of TiO_2_/PANI nanocomposites for the photocatalytic degradation of MNZ across six successive cycles under UV and visible light.^46^ After each degradation cycle, the composites were separated by centrifugation and used for the next cycle without any purification. The results showed degradation efficiencies of 98% and 96% after the first cycle and 86% and 85% after the sixth cycle under UV and visible light, respectively. In another study, Prasetyo et al. analyzed the recyclability of TiO_2_@PANI composites by decanting all remaining liquid from one cycle, while keeping the composites in the reactor, and refilling it with the same amount and concentration of CIP solution for the next cycle.^55^ No significant changes in the degradation efficiency of the composites toward CIP were observed after five cycles. Meanwhile, de Escobar et al. reused MIP25 across six cycles of photocatalytic degradation of DCF.^78^ At the end of each cycle, the composite solution was centrifuged for 15 min at 3500 rpm to remove the supernatant. Between two consecutive cycles, MIP25 was regenerated to remove the template DCF molecule. After six cycles of reuse, MIP25 exhibited almost the same degradation efficiency toward DCF, despite a noticeable loss in the adsorption capacity.

Other authors did not employ any composite regeneration procedure to study the reusability of synthetic polymer/TiO_2_ composites. For example, Zhou et al. assessed the reusability of the PBF–TiO_2_ nanocomposites by conducting three cycles of degradation experiments using aqueous solutions of NOR and MOX.^41^ The results showed that PBF–TiO_2_ nanocomposites maintained a degradation rate of >80% after three reuse cycles. In another work, Zhang et al. investigated the reusability of the TiO_2_/ONLH composites for the degradation of PRL under natural sunlight.^51^ After four successive cycles, the PRL degradation efficiency was relatively stable and reached 95.2% in the fourth cycle. In another study, the reusability of NF TiO_2_@PVDF nanocauliflower-like membranes was evaluated by performing three cycles of CIP photodegradation.^52^ The degradation efficiency of the membrane (above 90%) showed no significant reduction, even after three cycles. Meanwhile, Hasi et al. tested the stability of PVDF-CMPs–HS–S/TiO_2_ composites with four cycles of TC photodegradation, and the degradation efficiencies reduced from 90% after the first cycle to 89%, 88%, and 85% for the second, third, and fourth cycles, respectively.^63^ In another study, Sanches-Simões et al. reused TiO_2_@PU composites over five cycles of MNZ degradation and reported a negligible decrease in photocatalytic activity after the fifth cycle.^47^ Evgenidou et al. showed that the reuse of PET/TiO_2_ catalytic beads for the degradation of PGB was limited to two consecutive cycles.^89^ The photocatalytic rate constants were similar for the two cycles, but after the second cycle, the composite beads began losing their original shape, leading to a significant reduction in the TiO_2_ content.

Overall, the synthetic polymer/TiO_2_ composites demonstrated acceptable reusability, except for the system in which PET/TiO_2_ beads could be reused only for two consecutive cycles.^89^ At the same time, PP/TiO_2_ beads showed superior reusability, with a degradation efficiency of 90% even after 30 cycles.^53^ However, as with natural polymer/TiO_2_ composites, developing highly stable synthetic polymer/TiO_2_ composites that retain high pharmaceutical degradation performance is still an essential challenge in shifting to large-scale production and application of water cleaning materials.

#### 6.4.3 Reusability of the Hybrid Natural/Synthetic Polymer/TiO_2_ Composites

As mentioned above, rinsing with water is a simple method for regenerating polymer/TiO_2_ composites. Thus, Nguyen et al. investigated the separation and reuse ability of synthesized TiO_2_–AP1–0.4 composite beads across six consecutive cycles of OTC and ENR degradation (Figure 14A).^57^ After each cycle, TiO_2_–AP1–0.4 was separated from the solution by using sedimentation and filtration with filter paper, followed by multiple washes with distilled water before the next cycle. After six consecutive cycles, TiO_2_–AP1–0.4 hydrogel beads demonstrated good stability and excellent photocatalytic performance for both OTC and ENR, with degradation efficiencies of 87.7% and 79.5%, respectively.

**Figure 14:**
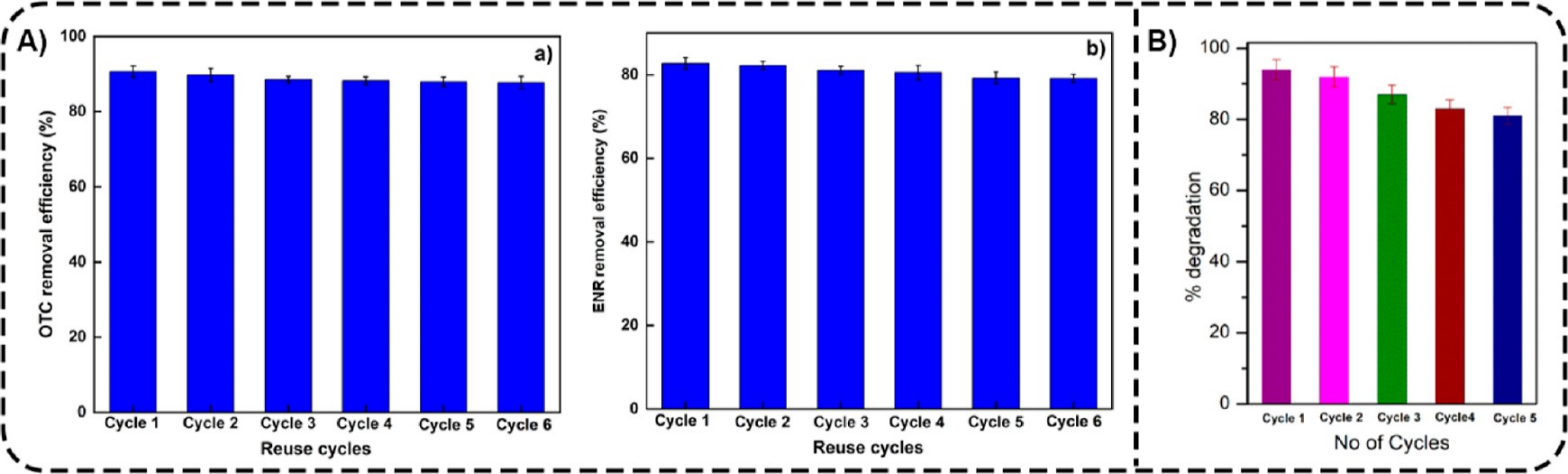
(A) Reusability of TiO_2_–AP1–0.4 hydrogel beads over six cycles of photocatalytic degradation of OTC (a) and ENR (b). Adapted with permission from the ref 57 Copyright 2024 Springer Nature. (B) Photocatalytic degradation of CIP over five consecutive cycles of composite reuse. Adapted from the ref 56 Copyright (2025), with permission from Elsevier.

Meanwhile, Pradhan et al. performed five consecutive cycles of CIP degradation using GG-*g*-PMAc-@-TiO_2_ QDs.^56^ The results showed that the degradation efficiency decreased to approximately 81% after the fifth cycle from 94% after the first cycle (Figure 14B). At that time, Neghi and Kumar explored the reusability of PVA–CS–TiO_2_ composites for three consecutive cycles of MNZ, CEF, and TC degradation.^39^ No changes in the photocatalytic activity of the composites were observed. In another study related to that mentioned above (Section 6.4.1. Reusability of the Natural Polymer/TiO_2_ Composites), Neghi et al. demonstrated excellent reusability of the PVA–CS–TiO_2_ composites, which showed complete MNZ removal and structural integrity after 15 photocatalytic cycles (Figure 12C).^45^

In summary, the composite systems of hybrid natural/synthetic polymer/TiO_2_ showed reusability comparable to that of natural polymer/TiO_2_ and synthetic polymer/TiO_2_ composites.

### 6.5 Effect of Real Water Systems on Pharmaceutical Removal

The removal efficiency of pharmaceuticals using polymer/TiO_2_ composites can be significantly affected by environmental conditions. In most cases, polymer/TiO_2_ composites have demonstrated highly efficient pharmaceutical removal in laboratory studies. However, their performance in real water systems and wastewater may change due to the compounding effects of various environmental factors, such as temperature, pH, ionic strength, and salinity, as well as the presence of other contaminants in wastewater. Therefore, to evaluate the prospects for the broad practical application of polymer/TiO_2_ composites, their effectiveness in pharmaceutical removal should be assessed in more complex real-water systems.

Kanmaz et al. used tap and drinking water, containing various ions and organic substances, to examine the effect of the water media on TC removal efficiency (Figure 15A).^64^ It was shown that the removal of TC from drinking and tap water was significantly lower than that from distilled water due to the presence of ions in these water media. A similar effect was observed in another study, wherein Raikar et al. investigated the photocatalytic degradation of CIP in distilled water, simulated groundwater (SGW), and municipal tap water.^53^ The degradation efficiency of CIP in SGW and real tap water was found to be about 74.6% and 94.6%, respectively, which were lower than that in distilled water (almost complete degradation) (Figure 15B). This could be caused by the presence of deactivating water constituents, such as Cl^–^, HCO_3_^–^, and natural organic matter. Additionally, the authors demonstrated that individual water constituents (Cl^–^, HCO_3_^–^, and humic acid) quench CIP degradation in a decreasing order: HA > Cl^–^ ≥ HCO_3_^–^. Since the deactivating water constituents in real tap water were lower than those in SGW, there were fewer negative impacts on CIP degradation.

**Figure 15:**
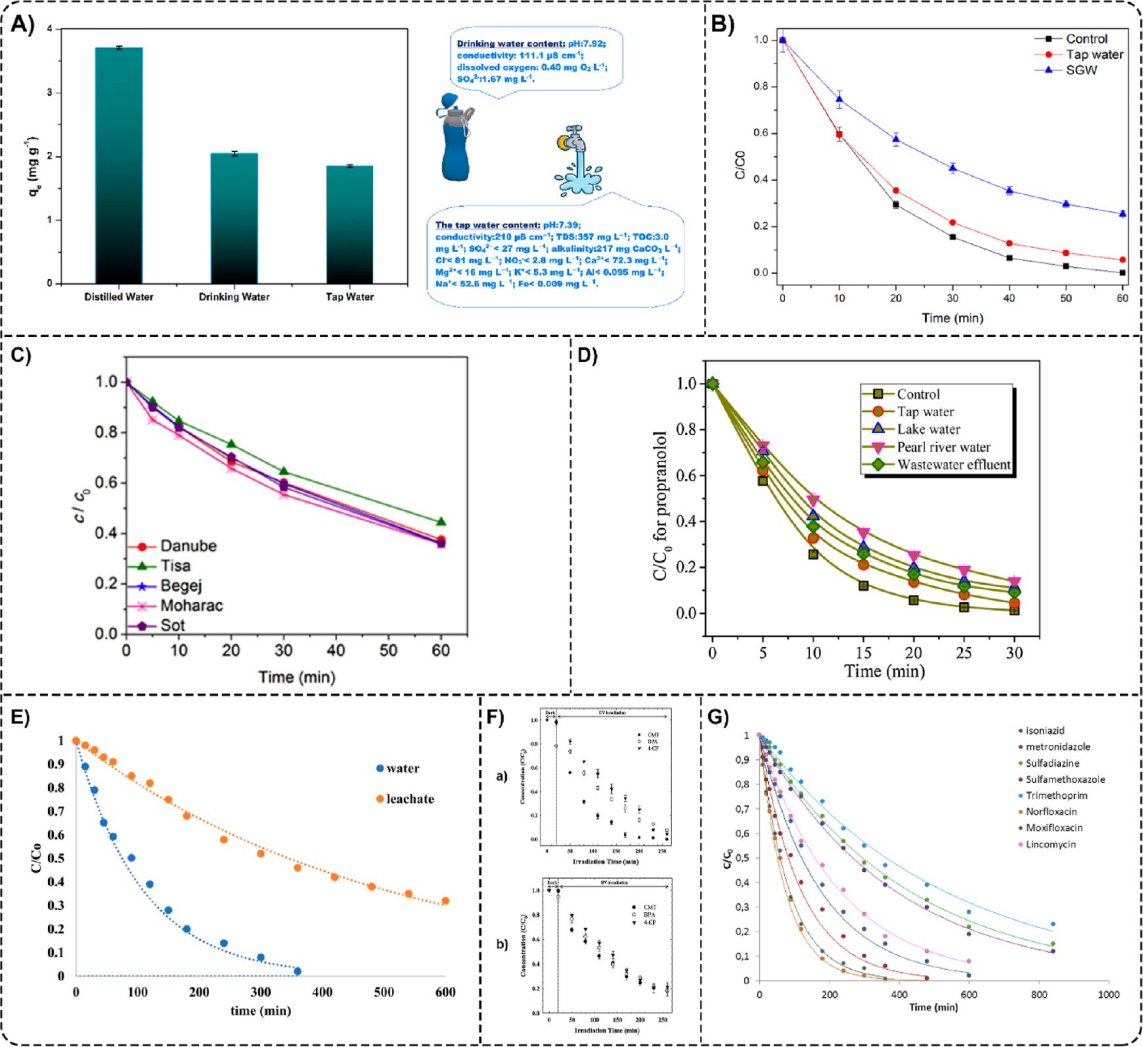
(A) Adsorption capacities toward TC in various water media. Reprinted from the ref 64 Copyright (2023), with permission from Elsevier. (B) Photocatalytic degradation of CIP in a real water matrix using PP/TiO_2_ beads. Reproduced from the ref 53 with permission from the Royal Society of Chemistry. (C) Photocatalytic degradation kinetics of APL with TP-100 composites in environmental waters and under UV exposure. Adapted from the ref 90 Copyright (2018), with permission from Elsevier. (D) Photocatalytic degradation of PRL in actual water/wastewater systems using TiO_2_/ONLH nanocomposites. Adapted from the ref 51 Copyright (2021), with permission from Elsevier. (E) Photocatalytic degradation of PGB in water and leachate using PET/TiO_2_ 30 wt % composite beads. Reprinted from the ref 89 Copyright (2023), with permission from Elsevier. (F) Adsorption under darkness (20 min) and photocatalytic degradation of CMT dissolved in secondary wastewater effluents (SWEs) from municipal wastewater treatment plant (MWWTP) (a) and institutional wastewater treatment plant (IWTP) (b), and under UV light with PVDF-TiO_2_-40 composites. Reprinted from the ref 87 Copyright (2015), with permission from Elsevier. (G) Photocatalytic degradation of antibiotics in wastewater effluent using PET-TiO_2_ composite films containing 10 wt % TiO_2_. Reprinted from the ref 40 Copyright (2019), with permission from Elsevier.

Environmental bodies of water, such as rivers and lakes, are another real water matrix explored in the degradation of pharmaceuticals by using polymer/TiO_2_ composites. For example, Šojić Merkulov et al. studied the photocatalytic degradation of APL using TP-100 composites in rivers (Danube, Tisa, and Begej) and lakes (Moharac and Sot) in Serbia (Figure 15C).^90^ The results indicate that the degradation efficiency of the composites toward APL was higher in environmental waters than in double-distilled water (45%). After 60 min of irradiation, the degradation efficiencies were 56% and 62% in Tisa and Danube water, while in the cases of Begej, Moharac, and Sot, they were 64%. The increased degradation efficiencies in rivers and lakes could be attributed to their higher pH values, as the initial pH of APL in double-distilled water was approximately 3.5, whereas in environmental waters it ranged from 7.6 to 8.0. In addition, the presence of SO_4_^2–^ could be another reason for the higher degradation efficiency of the composites toward APL in environmental waters. In another study, Zhang et al. investigated the degradation of PRL with TiO_2_/ONLH composites using tap water, wastewater effluent, ambient river water, and lake water (Figure 15D).^51^ After 30 min, the degradation of PRL was 98.7%, 95.5%, 88.9%, 86.1%, and 90.9% in pure water, tap water, lake water, Pearl River water, and wastewater effluent, respectively. Moreover, reduced removal efficiencies in other water systems compared to pure water were attributed to the presence of TOC, NO_3_^–^, and Cl^–^, which could inhibit photocatalyst activity by quenching free radicals or by photoshielding.

Evgenidou et al. studied the performance of PET/TiO_2_ 30 wt % composites during the degradation of PGB in percolated leachate, and the results showed slower photocatalytic degradation of PGB in the leachate than in water.^89^ Therein, less than 70% of PGB was degraded after 10 h of irradiation in the leachate, while the degradation efficiency in water was >98% after 6 h (Figure 15E). It was found that the half-life of PGB increased almost 5-fold, from *t*_1/2_ = 77.0 min in water to *t*_1/2_ = 346.6 min in leachate. The reason for this was attributed to the high content of inorganic and organic constituents in the leachate water, which act as scavengers for the generated radicals and reduce the frequency of photocatalytic events. In addition, the high concentration of organic matter and water color led to a light-shielding effect, reducing the photocatalyst’s efficiency.

Wastewater effluent, collected from wastewater treatment plants, hospitals, or industrial enterprises, appears to be the most valuable source of water for studying pharmaceutical removal using polymer/TiO_2_ composites. Ramasundaram et al. examined the photocatalytic degradation of CMT with PVDF–TiO_2_–40 composites in SWEs, one from an IWTP located at the Korea Institute of Science and Technology, and the other from a MWWTP located in Seoul (Figure 15F).^87^ In SWEs, significant kinetic retardation of photocatalytic degradation by the PVDF–TiO_2_–40 composites was observed. The reduction in photocatalytic efficiency was more pronounced for IWTP SWE with a higher organic matter concentration, confirming SWE’s radical-scavenging behavior due to its organic constituents. Meanwhile, Malesic-Eleftheriadou et al. investigated the degradation performance of PET-10 wt % TiO_2_ composite films toward a mixture of INH, MNZ, SDZ, SMZ, TMP, NOR, MOX, and LIN in effluent obtained from an urban wastewater treatment plant.^40^ The results showed the high efficiency of PET-10 wt % TiO_2_ composite films in photocatalytically degrading NOR, MOX, MNZ, INH, and LIN (more than 90%) within 10 h, SDZ and SMZ (more than 80%), and TMP (more than 70%) within 14 h. However, achieving these efficiencies in wastewater effluent required longer irradiation times than those in distilled water (Figure 15G). The slower degradation kinetics were attributed to organic and inorganic constituents in the effluent, which consumed a significant fraction of the formed radicals and thus competed with the antibiotic degradation. Moreover, organic and inorganic matter in the water can adsorb onto the catalyst’s surface, reducing the number of available active sites that interact with target pharmaceuticals. In another study, Farhadian et al. used a hospital’s effluent to investigate the photocatalytic degradation of TC by NST/CS nanocomposites.^70^ The nanocomposite’s degradation efficiency toward TC in the hospital effluent was 76.6%, which was lower than that in distilled water (91%) since the organic and inorganic constituents in the effluent consumed reactive species and lowered the composite’s antibiotic degradation efficiency.

Overall, the photocatalytic performance of polymer/TiO_2_ composites in real-world scenarios such as tap water, drinking water, rivers, lakes, and wastewater effluents was inferior to that in distilled water, except for the study by Šojić Merkulov et al.^90^ This trend was primarily attributed to the presence of organic and inorganic water constituents, which could inhibit the photocatalytic activity of composites by quenching the generated radicals or by having a photoshielding effect. These constituents could also be adsorbed on the photocatalyst’s surface, reducing the number of available active sites with which the target pharmaceuticals interact, which decreases their degradation efficiency.

## 7 Conclusions and Future Perspectives

The quality and availability of water are among the major high-priority concerns affecting the safety of water consumption and the global population’s standard of living. Among the water pollutants, pharmaceuticals, such as antibiotics, NSAIDs, anticonvulsants, and beta-blockers, have garnered increasing attention as emerging contaminants. Developing new strategies and materials, while improving existing ones, is a crucial challenge in water remediation. Polymer/TiO_2_ composites offer an alternative approach to designing materials for pharmaceutical removal and represent new, effective, and practical strategies for purifying contaminated water.

This review provides a comprehensive summary of polymer/TiO_2_ composites that have been widely explored for the removal of pharmaceuticals from water. Natural polymers, such as cellulose, chitosan, and alginate; synthetic polymers, such as PVP, PVDF, PES, and PANI; and hybrid natural/synthetic polymers can serve as TiO_2_-supporting materials for the fabrication of polymer/TiO_2_ composites that effectively remove pharmaceuticals. The reviewed studies have demonstrated various composite forms that are beneficial for meeting specific application requirements, and the main factors that contribute to differences in the composites’ photocatalytic performances include the amount of composite used, TiO_2_ loading, pH of the water, irradiation type, contact time, pharmaceutical concentration, pharmaceutical type, presence of inorganic and organic water constituents, and others. Moreover, this review bridges the gap in the field of polymer/TiO_2_ composites for water remediation by addressing key challenges associated with their industrial applications, including the toxicity of degradation products generated during photocatalysis, the stability and reusability of composite materials, and their performance in real-world water systems. Several studies have emphasized the importance of assessing the toxicity of polymer/TiO_2_ composites and the byproducts generated during the photocatalytic degradation of pharmaceutical contaminants, with the aim of minimizing the environmental impacts associated with their production and application. However, these studies were limited to models with a few living organisms or cell lines, or relied on computational and theoretical models, and the translation of these toxicity assessments to real environmental conditions has not been fully discussed. Some studies reported the regeneration and removal efficiencies of polymer/TiO_2_ composites across multiple reuse cycles, but these studies were limited to only five-six cycles, with some exceptions that demonstrated superior reusability for 15–30 cycles. The photocatalytic performance of polymer/TiO_2_ composites under real-world conditions was evaluated by using tap water, drinking water, river water, lake water, and wastewater effluents, and the results showed that the performance in these water systems was inferior to that in distilled water.

Overall, the use of polymer/TiO_2_ composites is considered a sustainable, reliable, and economical approach, and this review provides a more comprehensive understanding of their potential for water remediation. However, there are still immediate challenges in the field that need to be addressed for advancing polymer/TiO_2_ composites toward industrial water treatment applications, and future studies should be focused on the following aspects:

(1) The utilization of natural sunlight irradiation is suggested as a viable approach. Currently, most studies use UV and visible light irradiation (Tables 3–9), which account for only about 3–5% and 42–43% of naturally available sunlight, respectively.^203^ Consequently, removal efficiencies toward target pharmaceuticals under sunlight in real-world applications may vary significantly. Utilizing natural sunlight for the photocatalytic removal of pharmaceuticals in water treatment is essential for large-scale water remediation. It shows potential to improve the energy efficiency of the water-cleaning process and to promote widespread adoption of this approach.
(2) Real wastewater must be used for pharmaceutical removal studies because it contains inorganic and organic water constituents (e.g., inorganic ions, dyes, heavy metals, and humic acids), which can inhibit the photocatalytic activity of polymer/TiO_2_ composites. Multiple environmental factors significantly alter the removal of target pollutants in real-world scenarios compared to laboratory studies.
(3) Toxicity assessment of polymer/TiO_2_ composites and byproducts generated during photocatalytic degradation of pharmaceuticals is necessary to establish their safe and sustainable use. Developing nontoxic polymer/TiO_2_ composites with high pharmaceutical degradation efficiency is crucial to mitigate ecological risks. This concern is significant for synthetic polymers, whose low susceptibility to environmental degradation is advantageous for long-term use but can also lead to the formation and accumulation of microplastics, posing additional environmental hazards. It is worth noting that the toxicity of reaction mixtures can be determined by the type and concentration of the target pharmaceuticals and composites used, as well as the irradiation time.
(4) There are limited theoretical and computational studies to improve the predictability of photocatalytic performance of polymer/TiO_2_ composites without conducting additional time- and energy-consuming experiments. Some authors showed good agreement between experimental and predicted values of the photocatalytic degradation efficiency.^85^ In other studies, the authors used ECOSAR analysis to evaluate the toxicity of transformation products.^51,89,91^ These theoretical approaches should be further explored in the field of water-cleaning materials to improve the photocatalytic performance of polymer/TiO_2_ composites.
(5) Engineering highly stable and reusable polymer/TiO_2_ composites that do not compromise their photocatalytic performance for pharmaceutical removal remains an essential challenge for their practical applications, from both environmental and economic perspectives, since industrial water remediation requires efficient, durable, safe, and cost-effective materials. Nevertheless, alternative strategies, for example, using biodegradable polymer matrices that do not require recovery or reuse, should also be explored for real-world applications.
(6) Collaborative studies involving research scientists, data analysts, and manufacturing engineers would promote the development of polymer/TiO_2_ composites designed for large-scale production. The studies reported in this review have been conducted in a controlled laboratory environment, and practical considerations such as material selection, scalability, and long-term durability, which affect manufacturing design and cost-effectiveness, have not been thoroughly considered. Interdisciplinary research would also promote emerging material design approaches, such as AI-assisted designs.
